# The effect of dorsal column lesions in the primary somatosensory cortex and medulla of adult rats

**DOI:** 10.1016/j.ibneur.2023.05.005

**Published:** 2023-05-14

**Authors:** Atanu Datta

**Affiliations:** National Brain Research Centre, Manesar, Gurugram, Haryana 122052, India

**Keywords:** Cervical dorsal column lesions, Adult rats, Plasticity, Primary somatosensory cortex, Medulla, Electrophysiology

## Abstract

Spinal cord injury is a devastating condition that haunts human lives. Typically, patients experience referred phantom sensations on the hand when they are touched on the face. In adult monkeys, massive deafferentations such as chronic dorsal column lesions at higher cervical levels result in the large-scale expansion of face inputs into the deafferented hand cortex of area 3b. However, adult rats with thoracic dorsal column lesions do not demonstrate such large-scale reorganization. The large-scale face expansion in area 3b of monkeys is driven by the reorganization of the cuneate nucleus in the medulla. The sprouting of afferents from the trigeminal nucleus to the adjacent deafferented cuneate nucleus is facilitated by close proximity and compactness of the medulla in primates. Previously, in adult rats with thoracic lesions, the cuneate nucleus was not deafferented and its functional organization was not explored. The extent of the deafferentation and the duration of the recovery period are two major factors that determine the extent of reorganization. Hence, higher cervical (C3-C4) dorsal column lesions were performed, which cause massive deafferentations, and physiological maps were obtained after prolonged recovery periods (3 weeks −18 months). In spite of the above, the expansion of the intact face inputs was not observed in the deafferented zones of the primary somatosensory cortex (SI) and medulla of adult rats. The deafferented forelimb and hindlimb representations in SI were unresponsive to cutaneous stimulation of any part of the body. The cuneate and gracile nuclei in rats with complete dorsal column lesions remained mostly inactive except for a few sites which responded to stimulation of the spared upper arm. Hence, dorsal column lesions have different effects on the adult primate and rodent somatosensory systems. Appreciating this inter-species difference can aid in identifying the underlying neural substrates and restrict maladaptive reorganizations to cure phantom sensations.

## 1 Introduction

Spinal cord injury results in deafferentation and subsequent cortical reorganization which leads to the phenomenon of phantom sensation in a subset of human patients (Leemhuis and Gennaro, 2019, Moore et al., 2000). Understanding the neurobiological basis of adult plasticity in the somatosensory system in animal models is crucial for recovery from such injuries. Previous studies have used mainly non-human primates as the model system to study spinal cord injuries and very little is known of similar injuries and subsequent plasticity and recovery in rodents. In both primates and rodents, the perception of touch is mediated by the somatosensory system. Peripheral receptors on the skin transduce mechanical stimuli into neural signals and transmit them via the dorsal root ganglion or trigeminal ganglion to the medullary somatosensory nuclei.

In the rat, the gracile nucleus receives ascending afferents from the ipsilateral hindlimb, trunk, and tail, whereas the cuneate nucleus receives inputs from the ipsilateral forelimb, shoulder, and neck. The trigeminal sensory nucleus of rats receives inputs from the face and whiskers (Nord, 1967, Tracey, 2004, Waite, 2004). From the medulla, information pertaining to touch is relayed via the thalamus [Ventral posteromedial nucleus (VPM) and Ventral posterolateral nucleus (VPL)] to the SI. The VPM of the thalamus receives tactile inputs from the face (trigeminal nucleus) and the VPL receives tactile information from the forelimb and hindlimb (Francis et al., 2008, Lund and Webster, 1967a, Lund and Webster, 1967b). The SI is marked by the presence of a prominent whisker barrel field caudolaterally, jaws rostrolaterally, and forepaw and hind paw representations medially (Chapin and Lin, 1984, Pearson et al., 1996, Waters et al., 1995). The secondary somatosensory cortex (SII) is located lateral to the SI which contains a complete representation of the whole body surface (Benison et al., 2007, Santiago et al., 2019).

Peripheral or central perturbations lead to changes in the normal somatotopic arrangement at all three levels of the somatosensory system (Kaas et al., 2008). A number of studies have elucidated the guiding principles behind the organization of the brain and explored the extent and limits of plasticity resulting from a variety of injury-induced reorganizations in infant and adult brains (Dawson and Killackey, 1987, Pearson et al., 1999, Pons et al., 1991, Sengelaub et al., 1997). In monkeys and rats, manipulations of the periphery such as amputation of a digit, dorsal rhizotomy, or dorsal column lesions lead to alterations in these somatotopic maps (Halder et al., 2018, Jain et al., 1995, Kambi et al., 2014, Lane et al., 1995, Sengelaub et al., 1997). Cortical reorganization of rats is paralleled by changes in the somatotopy of the subcortical somatosensory nuclei of the thalamus and medulla (Li et al., 2014, Li et al., 2013, Pearson et al., 2003, Pearson et al., 1999).

Anatomical and electrophysiological studies in rats have shown that dorsal rhizotomy or forelimb amputation results in the expansion of inputs from the gracile nucleus into the deafferented cuneate nucleus of the medulla (Lane et al., 1995, Sengelaub et al., 1997). At the level of the thalamus, forelimb amputation induced the expansion of shoulder inputs into the deafferented forepaw representation of VPL of rats (Li et al., 2014). In adult rats that had undergone forelimb amputations, it has been demonstrated that regions of the deafferented forepaw barrel subfield (FBS) in the cortex started responding to stimulation of the shoulder (Pearson et al., 1999). Similarly, Lane et al. reported that in rats with forelimb amputations, the forepaw representations of the SI had abnormal receptive fields on the shoulder and the stump (Lane et al., 1995). These studies indicate that the somatosensory system of rats is capable of reorganization following peripheral injuries.

It has been reported in monkeys that dorsal column lesions between the levels of C2-C4 lead to the expansion of intact face inputs into the deafferented hand area in the SI (Jain et al., 2008). A number of studies on extensive long-term deafferentations have suggested that the proposed mechanism underlying such large-scale reorganizations in monkeys involves the sprouting of intact afferents from the trigeminal nucleus into the deafferented cuneate nucleus at the level of the medulla (Jain et al., 2008, Jain et al., 2000, Pons et al., 1991). Furthermore, it has been demonstrated that the large-scale expansion of intact inputs from the face into the deafferented hand cortex of area 3b in monkeys is due to reorganization at the level of the cuneate nucleus of the medulla and not because of a cortico-cortical mechanism (Chand and Jain, 2015, Halder et al., 2018, Kambi et al., 2014). The inactivation of the reorganized cuneate nucleus by lidocaine abolished the expansion of face inputs in the reorganized hand representation of area 3b in the cortex. This study also ruled out thalamic plasticity in the VPL to be the driver of cortical reorganization in area 3b of monkeys (Kambi et al., 2014). However, in rats with dorsal column lesions at the level of the thoracic vertebra (T6-T8), the deafferented hind paw representation in the SI remained inactive and was not substituted by the intact forelimb inputs in the SI (Jain et al., 1995).

Somatotopic maps are represented within a much smaller neural space in the brainstem (Pons et al., 1991). The close proximity of the trigeminal nucleus to the cuneate nucleus at the level of the medulla helps in the sprouting of inputs from the trigeminal nucleus to the adjacent deafferented cuneate nucleus of monkeys over a prolonged recovery period following dorsal column lesions at high cervical levels (C3-C5) (Jain et al., 2000) which in turn drives the large scale reorganization in area 3b of monkeys (Kambi et al., 2014). Furthermore, in the previous study, thoracic dorsal column lesions in rats did not deafferent the cuneate nucleus and the extent of reorganization at the level of the medulla of adult rats was not explored (Jain et al., 1995). Therefore, we performed comparable unilateral lesions of dorsal columns at cervical (C3-C4) levels of adult rats to deafferent the cuneate nucleus and unravel the extent of reorganization at the level of the SI and the somatosensory nuclei at the level of the medulla after long periods of recovery.

## 2 Methods

The procedures performed on all animals used in this study were approved by the Institutional Animal Ethics Committee (IAEC) of the National Brain Research Centre, and the Committee for the Purpose of Control and Supervision of Experiment in Animals (CPCSEA), Government of India. During the course of the study animals had ad libitum access to food and water. A total of twenty-six adult male Long Evans rats were used for the study. Of these, four animals each served as normal controls for the cortex and medulla (that is, n = 8), to determine the somatotopic organization within these brain regions. The dorsal funiculus was lesioned unilaterally at cervical levels (C3-C4) in adult rats and after a recovery period ranging from three weeks to eighteen months (for details see Table 1), they were investigated for the extent of reorganization. The effect of dorsal column lesions on the organization of the SI was studied in eleven animals. The somatotopy of the cuneate and gracile nucleus was determined in fourteen animals, after partial or complete dorsal column lesions, by standard multiunit mapping. In seven of these experiments, the cortex and medulla were mapped in the same animal in order to elucidate the relationship between the organization of the somatosensory nuclei of the medulla and that of the SI. The details of each animal are provided in Table 1.

**Table 1:** Details of the animals used for the study.

Rat ID	Area mapped	Lesion remarks	Weight during lesion surgery (gm)	Recovery period (days)	Weight During Mapping (gm)	Recording sites (Cortex)	Recording sites (Medulla)
16-04RT	Cortex	Normal	–	–	452	67	–
20-86RT	Cortex	Normal	–	–	360	75	–
15-136RT	Cortex	Normal	–	–	530	72	–
14-29RT	Cortex	Normal	–	–	450	88	–
16-109RT	Cortex	Complete	350	49	350	52	
17-19RT	Cortex	Incomplete	470	521	475	49	
17-18RT	Cortex	Incomplete	455	507	430	61	
17-09RT	Cortex	Incomplete	500	22	450	48	
20-103RT	Medulla	Normal	–	–	497		135
20-87RT	Medulla	Normal	–	–	425		141
15-90RT	Medulla	Normal	–	–	560		207
15-98RT	Medulla	Normal	–	–	580		186
20-101RT	Medulla	Complete	490	162	490		137
20-91RT	Medulla	Complete	465	197	470		146
20-92RT	Medulla	Complete	465	229	475		116
20-93RT	Medulla	Complete	540	251	515		70
20-99RT	Medulla	Complete	470	145	370		85
17-16RT	Medulla	Incomplete	520	488	630		55
18-79RT	Medulla	Incomplete	480	268	430		118
17-54RT	Cortex, Medulla	Complete	515	196	480	50	104
20-96RT	Cortex, Medulla	Complete	500	278	455	36	23
20-97RT	Cortex, Medulla	Complete	460	288	400	50	91
20-98RT	Cortex, Medulla	Complete	440	309	430	52	99
17-37RT	Cortex, Medulla	Incomplete	490	160	560	47	65
17-65RT	Cortex, Medulla	Incomplete	350	157	430	51	89
17-20RT	Cortex, Medulla	Incomplete	450	555	564	50	53

### 2.1 Dorsal column lesions

Long Evans rats were administered dexamethasone (2 mg/kg, IM) and glycopyrrolate (6 µg/kg, IM) before the surgery to prevent inflammation and excessive salivation. This was followed by anesthetizing the rats with ketamine hydrochloride (80 mg/kg, IM) and xylazine hydrochloride (7 mg/kg, IM). Supplementary doses of ketamine were administered to maintain the anesthesia, as required throughout the experiments. An incision was made along the midline from the base of the skull till the fifth cervical vertebra. After laminectomy of the cervical vertebra (C3-C4), the dura mater was incised to expose the spinal cord. Subsequently, the dorsal column was lesioned unilaterally with a pair of fine forceps (Supplementary Fig. 1A-B). A lesion at this level of the spinal cord was expected to deactivate the ascending afferents from forelimb, hindlimb, trunk, and tail while completely sparing facial inputs to the medulla. After suturing the muscle and skin, rats were administered an antibiotic (Enrofloxacin; 5 mg/kg, IM), an anti-inflammatory drug (dexamethasone; 0.2 mg/kg, IM) and an analgesic (Butrum; 0.2 mg/kg, IM) for the next five days. The animals were closely monitored during the post-surgical recovery period for any signs of autotomy.

### 2.2 Electrophysiological recordings

The aim of the present study was to explore large-scale reorganization in the deafferented representation of the forelimb, hindlimb and, trunk at the level of SI and medulla. Hence, electrophysiological techniques were employed to cover the large expanse of the SI and medulla as described in previous studies (Halder et al., 2018, Jain et al., 2008).

#### 2.2.1 Primary somatosensory cortex

The SI of rats that had undergone dorsal column lesions was explored with standard multiunit mapping procedures [as described in (Jain et al., 2008, Jain et al., 1995, Tandon et al., 2009)] after the aforementioned recovery period. Rats were anesthetized with a mixture of ketamine hydrochloride (80 mg/kg, IM) and xylazine hydrochloride (7 mg/kg, IM). The head was stabilized in a stereotaxic head holder (Kopf Instruments, CA, USA) and a craniotomy (Supplementary Fig. 1C) was performed over the SI contralateral to the dorsal column lesion. Tungsten microelectrodes (1 MΩ at 1 kHz; Microprobe, MD, USA) were used for electrophysiological recordings from a depth of 400–800 µm of SI. Electrode penetrations were placed in a pattern to carefully avoid blood vessels and to cover the forelimb, hindlimb, trunk, and other representations of SI. Penetration sites were marked on enlarged photographs of the exposed cortex. The forelimb, hindlimb, trunk, face, and other body surfaces were stimulated with fine brushes and blunt probes to evoke responses in SI and the corresponding skin surface was defined as the receptive field of that recording site. The responses evoked by light touch on the skin or hair were qualitatively classified as “cutaneous” or “hairy” and as “tap” responses if elicited by hard probing of the body surface (Jain et al., 1995). Detectable increase in neural activity, in response to tactile stimulation, was monitored auditorially by a speaker and visually by an oscilloscope (Hameg Instruments, HM 507, Germany). The receptive fields of each penetration site were drawn on the photograph of the appropriate body part of the animal. Recording sites that did not respond to stimulation of any of the body surfaces were designated as unresponsive sites. After completion of the recordings, small electrolytic lesions were made in the cortex to facilitate the reconstruction of the somatotopic maps (Supplementary Fig. 1D).

#### 2.2.2 Medulla

Animals were anesthetized (as discussed earlier) and the head was secured in the stereotaxic head holder. The skin was incised along the midline of the dorsal neck till the base of the skull. After removing the fascia, neck muscles were retracted layer by layer. The caudal end of the occipital bone of the skull was removed and the dura was retracted to expose the medulla near the obex (Supplementary Fig. 2A). Electrode penetration sites were marked on a magnified photograph of the exposed medulla. Microelectrode recordings were obtained from the cuneate, gracile, and trigeminal nucleus of the medulla ipsilateral to the spinal cord lesion. The electrode penetrations were placed in a grid-like pattern while carefully avoiding major blood vessels. The electrode was moved along the dorsoventral axis of the medulla using a hydraulic microdrive (David Kopf Instruments, CA, USA). At each recording site, the forelimb, hindlimb, trunk, face, and other parts of the body were stimulated with brushes or probes. Receptive fields were recorded at depths of every 50 µm from the surface of the medulla until neurons were unresponsive to the tactile stimulation of all body surfaces. This was followed by drawing the receptive fields on the photograph of the appropriate body part of the animal. Responses to different types of stimuli were recorded as described above, for the cortex. After the completion of microelectrode recording sessions, electrolytic lesions were made at some of the selected penetrations at sites ventral to the cuneate and gracile nucleus (Supplementary Fig. 2B-C). At some sites, lesions were made in a continuous fashion while retracting the electrode at a very slow speed (100 µm per second; Supplementary Fig. 2D-E). These provide information about the electrode angle and the depth of the recordings which were used to overlay the physiological map on histological sections of the medulla.

### 2.3 Histology

After completing recording sessions, animals were perfused transcardially with 0.01 M phosphate-buffered saline followed by 2 % paraformaldehyde and 2 % paraformaldehyde containing 10 % sucrose. The brain and spinal cord were carefully removed (Supplementary Fig. 1A). The cortex was flattened (Supplementary Fig. 1E) and all parts of the brain and spinal cord were stored in 30 % sucrose for cryoprotection. The flattened cortex was frozen and sectioned at a thickness of 60 µm in a plane parallel to the pial surface with the help of a sliding microtome and subsequently stained for cytochrome oxidase (CO) [Supplementary Fig. 1D; (Wong-Riley, 1979)]. The medulla was sectioned at 50 µm in the coronal plane and also stained for cytochrome oxidase to delineate nuclear boundaries and identify electrolytic lesions to align the physiological map with corresponding histological sections (Supplementary Fig. 2B-E). The spinal cord was sectioned in the horizontal plane at a thickness of 50 µm. Unstained sections of the spinal cord were mounted on a glass slide and the sites of the lesion were drawn using a dark field microscope and a camera lucida (Supplementary Fig. 1B).

### 2.4 Reconstruction of cortical maps

Somatotopic maps were reconstructed using Canvas software (version 10; ACD systems) as described previously (Jain et al., 1995). Each recording site on the enlarged brain picture was marked with specific symbols used to denote the type of stimulus used at that site. Responses from different parts of the body surface were delineated and grouped together with designated colors to visualize the somatotopy of the cortex. Thereafter, histological sections of the flattened cortex were overlaid on the physiological maps by aligning them with the electrolytic lesions (Supplementary Fig. 1D). In cases where the CO isomorph was spread across a few sections of the flattened cortex, outlines of the isomorph were drawn from multiple sections in the camera lucida by aligning them using the electrolytic lesions and the positions of major blood vessels as reference.

### 2.5 Reconstruction of somatotopic maps in medullary nuclei

Receptive fields at the recording sites of each penetration were reconstructed along the dorsoventral axis of the medulla using the Canvas software. The penetrations were first placed on sites marked on the photograph of the exposed medulla. Thereafter, they were overlaid onto the outline drawings of the corresponding coronal sections of the medulla [obtained from the photomicrographs acquired using a microscope (Leica DMRXA2)] and using the sites of electrolytic lesions as reference.

### 2.6 Reconstruction of the spinal cord

Outlines of horizontal sections of the spinal cord around the site of the lesion were drawn using a camera lucida to elucidate the extent of the lesions and the boundaries of white and grey matter. The drawings of each horizontal section were opened in the Canvas software and the coronal view of the spinal cord at the site of the lesion was reconstructed (Supplementary Fig. 1B).

### 2.7 Statistical analysis

Animals were grouped according to the extent of the dorsal column lesions into the normal, complete lesion (CL), and incomplete or partial lesions (ICL). The results obtained in our experiments did not correlate with the recovery periods of the animals, hence, they were grouped together on the basis of the lesion. The main goal of our study was to explore the large-scale expansion of face afferents into the deafferented forelimb, hindlimb, and trunk representation at the level of the SI and the medulla of animals with dorsal column lesions. Hence, recording sites were mainly classified into three main information processing streams or pathways, that is, forelimb, hindlimb and lower body, and face depending upon the receptive fields of the neurons at specific recording sites. Recording sites that had neurons with receptive fields on the forepaw, arm, and the upper arm near the shoulder were considered in the forelimb group. Similarly, the group labeled as the hindlimb and lower body included sites responsive to stimulation on the foot, heel, thigh, trunk, and tail. The sites that had receptive fields on the lower jaw, chin, upper lip, and lower lip, whiskers, and other parts of the face were assigned to the face group for statistical analysis. In each animal, the total number of forelimb responsive sites within the forelimb representation of SI was divided by the total number of recording sites (including non-responsive sites) in the forelimb representation to calculate the forelimb percentage site for each animal. For data visualization, the mean percentage ± standard error of the mean (SEM) was calculated for each group and compared. Similarly, the mean percentage of unresponsive sites for each group was calculated. This measure for non-responsive sites was calculated because dorsal column lesions in the rat somatosensory system lead to the inactivation of the somatosensory cortex, which is not reactivated by inputs from intact body parts even after prolonged recovery periods. Similar measures for the hindlimb and lower body and face were calculated for each animal in every group (normal or CL or ICL) for SI. The recording sites inside the cuneate nucleus that responded to stimulation of the forelimb, such as digits, pads, forepaw, forearm, upper arm, and shoulder, were calculated as a percentage of total recording sites located inside the cuneate nucleus. Hindlimb and lower body responsive sites, such as trunk and tail, were also represented by a percentage of the total recording sites inside the gracile nucleus. The face-responsive sites from the trigeminal nucleus in the medulla were calculated in a similar manner. The percentages of non-responsive sites within the dorsal column nuclei were calculated similarly.

All the statistical analyses were performed using SigmaPlot software (SYSTAT; version 14, USA). Data were first checked for normality and equal variance (homoscedasticity) by performing Shapiro-Wilk and Brown-Forsythe tests, respectively, before performing any statistical tests (ANOVA, correlation analysis, or t-test). If the data did not conform to the assumptions of one-way ANOVA (analysis of variance) a non-parametric Kruskal-Wallis test was performed to compare different groups. For data sets which were normally distributed, a one-way ANOVA was performed. A Holm-Sidak post-hoc test or Dunn’s post-hoc test was performed for multiple comparisons using SigmaPlot which generates adjusted P values for pairwise multiple comparisons. Therefore, an alpha level of 0.05 was used to report significance. A Mann-Whitney Rank Sum Test was performed to compare differences between the percentage of responsive sites in normal animals and animals with complete lesions for the face since only one data point was available for animals with incomplete lesions. The relationship between spared responses from lesioned animals in the medulla and SI was investigated by performing the Pearson product-moment correlation or Spearman rank order correlation test.

## 3 Results

A total of 26 adult Long-Evans rats were used for the study. Out of these, 18 animals had undergone dorsal columns lesion and after recovery periods ranging from 3 weeks to 18 months (Table 1) the SI and the dorsal medullary nuclei were mapped using standard multiunit electrophysiological techniques (Jain et al., 2008, Tandon et al., 2009). In 11 of these rats with dorsal columns lesion, receptive fields were recorded at 546 sites in the SI. Four animals served as normal control from which we obtained 302 recording sites. The results of the medulla are based on recordings of receptive fields from 1251 recording sites, from 187 penetrations in 14 lesioned animals. Similarly, 4 animals were used as control for medulla mapping from which 669 recording sites were obtained from 69 penetrations.

### 3.1 Topographic organization of the somatosensory cortex in normal rats

**Fig. 1:**
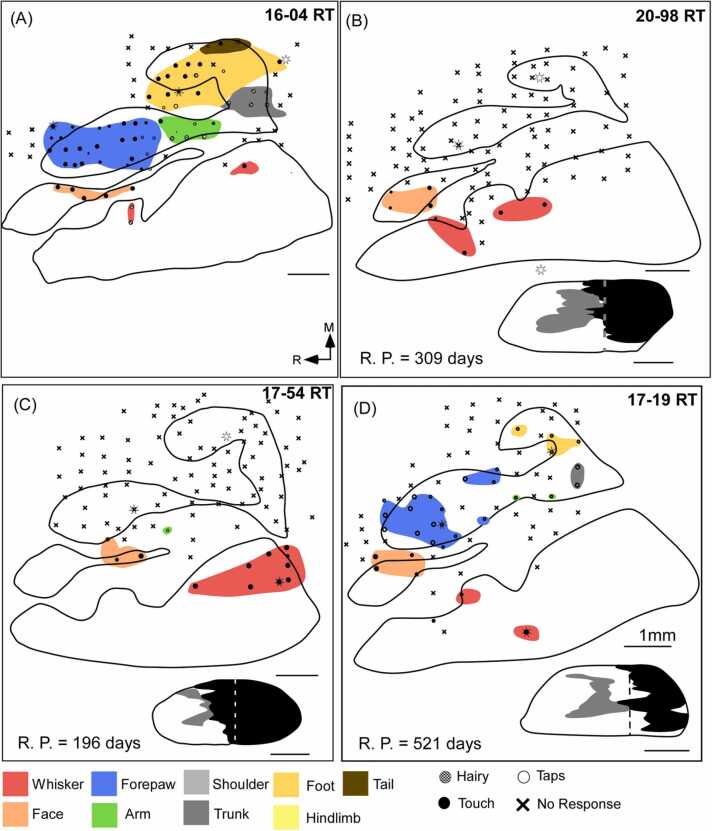
Electrophysiological maps of the primary somatosensory cortex in rats. The tracing of CO isomorphs and the electrophysiological maps were aligned based on microlesions (denoted by stars) to obtain somatotopic maps. Reconstructions of the spinal cord are shown below each map in the lesioned animals (A) A topographic organization of somatosensory receptive fields of the hindlimb, trunk, forelimb, forepaw, face, and whisker in a normal rat. (B) In a rat with the complete lesion of contralateral dorsal columns, as represented by the spinal cord reconstruction, the deafferented forelimb, hindlimb and trunk representations in the cortex are largely unresponsive to tactile stimulation of any body part. (C) However, in some animals such as 17–54 RT, only one site responsive to the upper arm has been observed. (D) In a rat with a partial lesion of the dorsal columns, a few responsive sites are observed due to sparing. Scale bar: 1 mm. M, medial; R, rostral; R. P., recovery period (post-lesion).

Electrophysiological mapping of the somatosensory cortex in normal rats (n = 4) demonstrated that somatosensory inputs from the different body parts were topographically organized in the SI of the rat cortex (Fig. 1A). The hindlimb representation is located most medially and spreads about ∼ 2 mm posterior to the bregma. The trunk representation is found lateral and caudal to the hindlimb cortex. The forepaw cortex (FBS) is located approximately 4 mm lateral from the midline and spreads approximately 3 mm anterior to the bregma. The arm representation was found caudal to the FBS. The region of the cortex lateral to the FBS responded to the tactile stimulation of the contralateral lower lip, and lower jaw (Fig. 1A). The whisker barrel cortex is found immediately lateral to this lower jaw representation. The overall somatotopy was found to be similar to the previously published studies (Chapin and Lin, 1984, Waters et al., 1995).

Neurons in the FBS responded to light touch on the digits and pads of the forepaw. The receptive fields comprised of tips of digits, whole single digits, pads, and occasionally two digits and palmer pads. Neurons of the arm representation responded to touch on the wrist, hairs, or skin of the arm (Fig. 1A and Supplementary Fig. 3A-C). The trunk representation mostly had neurons that responded vigorously to stimulation of hairs on the contralateral body surface. The hind paw cortex (HBS; hindpaw barrel subfield) responded to the tactile stimulation of the ventral foot pads, toes, and heel (Fig. 1A and Supplementary Fig. 3D).

### 3.2 Changes in the somatotopy of the somatosensory cortex following complete dorsal column lesions

Five rats were used to study the effects of complete lesions of the dorsal columns on the somatotopy of the SI. The forelimb ( % Responsive sites – N: 97.8092 ± 1.2459 % vs CL: 6.7914 ± 3.8899 %), hindlimb, and lower body ( % Responsive sites – N: 87.3397 ± 5.1698 % vs CL: 3.0769 ± 2.7521 %) representations were largely inactive, as demonstrated by the somatotopic map of 20–98 RT (Fig. 1B). Neurons of the deafferented forelimb and hindlimb cortex did not respond to the tactile stimulation of any other intact body surfaces such as those on the face and whiskers. However, in two of these animals 17–54 RT (Fig. 1C) and 20–97 RT (discussed later), single sites were encountered which had receptive fields for the upper arm near the shoulder. In 16–109 RT (Supplementary Fig. 4), three sites that were responsive to taps on the pads were found in the FBS, and two sites that responded to stimulation of the middle and the base of the tail were encountered at sites caudal to the HBS, probably as a result of a few spared fibers of the dorsal columns as observed in similar studies (Jain et al., 1995, Kambi et al., 2014). Neurons in the adjacent lower jaw representation responded vigorously to light touch on the lower lip and lower jaw vibrissae, respectively (Supplementary Fig. 3F-G). The face and whisker responses were found in their expected locations as observed in normal rats (Supplementary Fig. 3A and E). There was no expansion of inputs from the lower jaw, face, or whiskers into the deafferented forelimb and hindlimb cortex.

### 3.3 Alterations in the organization of the somatosensory cortex after incomplete lesions of the dorsal columns

The somatosensory cortex of six rats was examined using standard multiunit mapping techniques after a partial lesion of the dorsal columns (Fig. 1D). The cortex was composed of discontinuous patches of responsive (Forelimb: 65.1057 ± 7.061 %; Hindlimb and Lower body: 31.5899 ± 8.6214 %) and unresponsive sites (Forelimb: 34.8942 ± 7.061 %; Hindlimb and Lower body: 68.41 ± 8.6214 %). The responses were weak and mostly evoked by hard taps on the body surface. Weak responses to light touch were also observed at a few sites. There was no expansion of inputs from the lower jaw or whiskers or any other intact body parts which were spared due to the partial lesion of the dorsal columns. The number of responsive sites in the forelimb and hindlimb cortex was variable across animals and depended on the extent of the spinal cord lesion (Fig. 1D). The responses to chin and whisker stimulation were strong and were observed at their corresponding locations.

### 3.4 Analysis of responsive sites in the SI of normal and lesioned animals

**Fig. 2:**
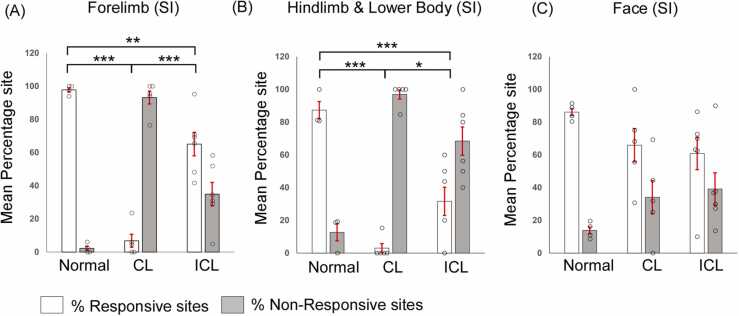
Comparison of recording sites in the primary somatosensory cortex (SI). Bar plots representing mean ± SEM of percentage responsive and non-responsive sites in (A) forelimb cortex, (B) hindlimb and lower body representation of SI, and (C) anterolateral barrel subfield and whisker barrel cortex of SI. In both forelimb (A) and hindlimb and lower body (B) the percentage of responsive sites in the complete lesioned animals are significantly lesser than the normal and incomplete lesioned rats. Moreover, the incomplete lesioned animals had a significantly lesser percentage of responsive sites than the normal group. No significant differences were observed across the experimental groups for the face-responsive sites. The hollow circles shown in the plots represent data points from individual rats. CL, complete lesion; ICL, incomplete lesion. *, P ≤ 0.05; **, P ≤ 0.01; ***, P ≤ 0.001.

A comparison of the percentage of sites responsive to stimulation of the forelimb between normal and lesioned animals demonstrated that there were significantly fewer forelimb-responsive sites in animals with complete and incomplete lesions compared to the normal animals (ANOVA, F_(2,12)_ = 53.388, P < 0.001; Holm-Sidak post hoc test, N vs CL, P < 0.001; ICL vs CL, P < 0.001; N vs ICL, P = 0.003; Fig. 2A). Additionally, animals with complete lesions had significantly fewer responsive sites than those with incomplete lesions. In rats with complete dorsal column lesion, hindlimb, and lower body percentage responsive sites were significantly fewer than those in normal and incomplete lesioned animals (ANOVA, F_(2,11)_ = 23.634, P < 0.001; Holm-Sidak post hoc test, N vs CL, P < 0.001; CL vs ICL, P = 0.017; Fig. 2B). Moreover, statistical differences were also observed between incomplete lesioned animals versus normal where post hoc analysis demonstrated that incomplete lesions had fewer responsive sites than those in normal animals (Holm-Sidak post hoc test, ICL vs N, P = 0.001; Fig. 2B). Furthermore, no statistical differences were observed in the percentage of sites responsive to stimulation of the face between the normal and lesioned animals (Fig. 2C).

### 3.5 Organization of the somatosensory nuclei in the medulla of normal rats

**Fig. 3:**
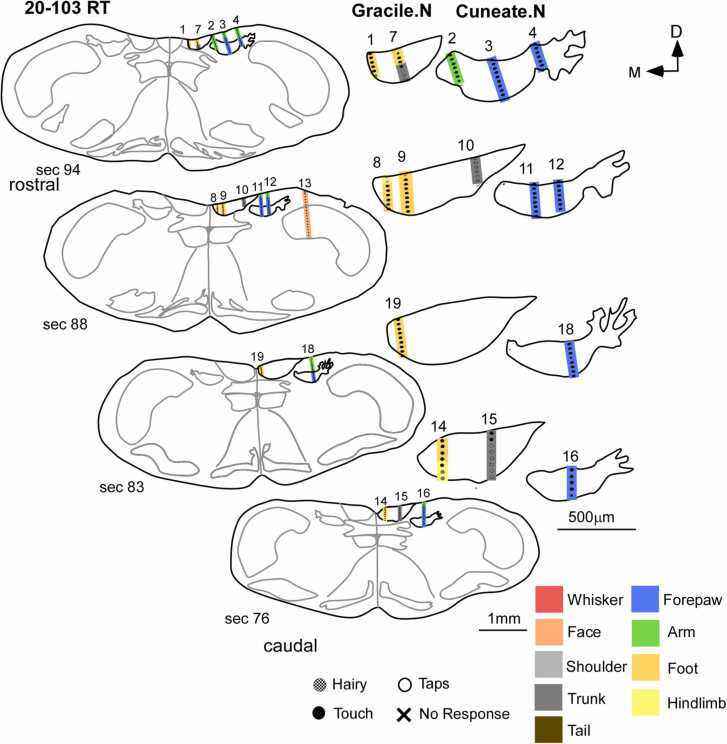
Organization of receptive fields in the somatosensory nuclei of the medulla in a normal rat. Schematics of coronal sections of the medulla (left) show the sites of electrode penetrations. A magnified view of these electrode penetrations in the gracile and cuneate nuclei of the corresponding sections is shown on the right. The sites in the gracile nucleus responded to tactile stimulation of the ipsilateral hindlimb, foot, and trunk. Receptive fields for the ipsilateral forelimb and forepaw are present in the cuneate nucleus, located lateral to the gracile nucleus. Neurons in the trigeminal nucleus, located lateral to the cuneate nucleus, respond to stimulation of the face. Scale bar:1 mm (whole section schematic), 500 µm (inset); D, dorsal; M, medial.

Somatosensory nuclei of the medulla, that is, the gracile, cuneate, and trigeminal nuclei are arranged in a medial to lateral pattern, respectively, in normal rats (n = 4) as reported in previous studies (Li et al., 2013, Nord, 1967). Briefly, responses were strong and mostly due to the light tactile touch on glabrous surfaces of the skin or stimulation of hair on the respective body surfaces. Responses in the gracile nucleus comprised of foot, hindlimb, trunk, and tail (Supplementary Fig. 5A and C). The neurons in the cuneate nucleus had receptive fields on digits, pads, and arm (Supplementary Fig. 5A-B). The receptive fields in the forepaw typically comprised of those corresponding to single or multiple digits and pads, also seen in earlier studies (Li et al., 2012). The receptive fields of recording sites in a penetration placed in the trigeminal nucleus progressed from the medial part of the lower jaw followed by those on the lower lip, corner of the mouth, and the upper lip, in a dorsal to ventral manner (Fig. 3 and Supplementary Fig. 5A and D).

### 3.6 Cuneate and gracile nucleus remains largely inactive after complete dorsal column lesions

**Fig. 4:**
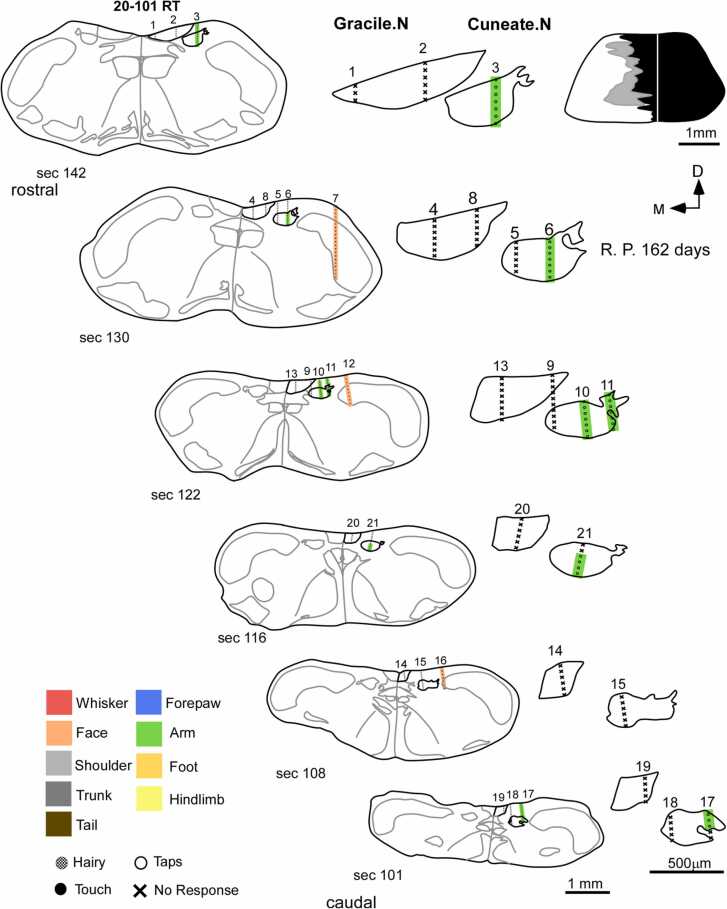
Organization of receptive fields in the medulla of a rat with complete lesion of dorsal columns. On the left, schematics of coronal sections of the medulla demonstrate sites of electrophysiological recording from the gracile, cuneate, and trigeminal nuclei in the case of a complete dorsal column lesion (schematic on the top right). Magnified schematics of the cuneate and gracile nuclei of the corresponding sections are displayed on the right. The gracile nucleus stays largely inactive and neurons at these recording sites do not respond to tactile stimulation of the foot, hindlimb, trunk, or any other intact body parts. The cuneate nucleus also remains inactive, except for a few sites wherein receptive fields of the upper arm near the shoulder were observed. Recording sites in the trigeminal nucleus follow the similar receptive field progression of sites on the face as observed in the medulla of a normal animal. There was no large-scale reorganization or expansion of intact chin inputs into the deafferented cuneate and gracile nucleus. Scale bar:1 mm (whole section schematic), 500 µm (inset); D, dorsal; M, medial; R. P., recovery period (post-lesion).

Nine rats were used to study the effects of complete lesions of the dorsal columns on the somatotopic organization of the cuneate and gracile nucleus. The deafferented cuneate and gracile nuclei remained largely inactive after complete dorsal column lesions and there was no expansion of intact face inputs into these nuclei. The cuneate nucleus appeared as a mosaic of responsive (48.2705 ± 8.8866 %) and unresponsive sites (51.7295 ± 8.8866 %). These responses were weak and evoked by hard taps delivered to the upper arm near the shoulder (Fig. 4 and Supplementary Fig. 5E-F). This was the same and only receptive field encountered at all recording sites in the penetrations made in the cuneate nucleus. Neurons in the gracile nucleus did not respond to stimulation of the foot, hindlimb, trunk, ( % Responsive sites – N: 90.2689 ± 4.4150 % vs CL: 3.4343 ± 3.2379 %; Fig. 4 and Supplementary Fig. 5E-F) or any other intact body parts like the medial lower jaw, lower lip or upper lip. However, in rat 17–54 RT (Supplementary Fig. 6A), weak responses to touch on the hindlimb, foot, trunk, and the tail were encountered in the gracile nucleus, probably due to spared fibers (Kambi et al., 2014). The electrode penetrations in the trigeminal nucleus demonstrated the same receptive field progression along the dorsoventral axis of the nucleus as was observed in the normal animal ( % Responsive sites – N: 92.7046 ± 2.5360 % vs CL: 96.53 ± 2.2142 %; Supplementary Fig. 5D and G). There was no expansion of trigeminal inputs like the medial part of the chin, lower jaw, or lip into the deafferented cuneate or gracile nucleus. Only in one animal (20–98 RT), we did not find any responses to touch on the upper arm near the shoulder in the cuneate (Supplementary Fig. 6C). This may have resulted from the rostral expansion of scar tissue around the site of lesions of the dorsal column (Supplementary Fig. 6D).

### 3.7 Effects of incomplete lesions on cuneate and gracile nucleus

**Fig. 5:**
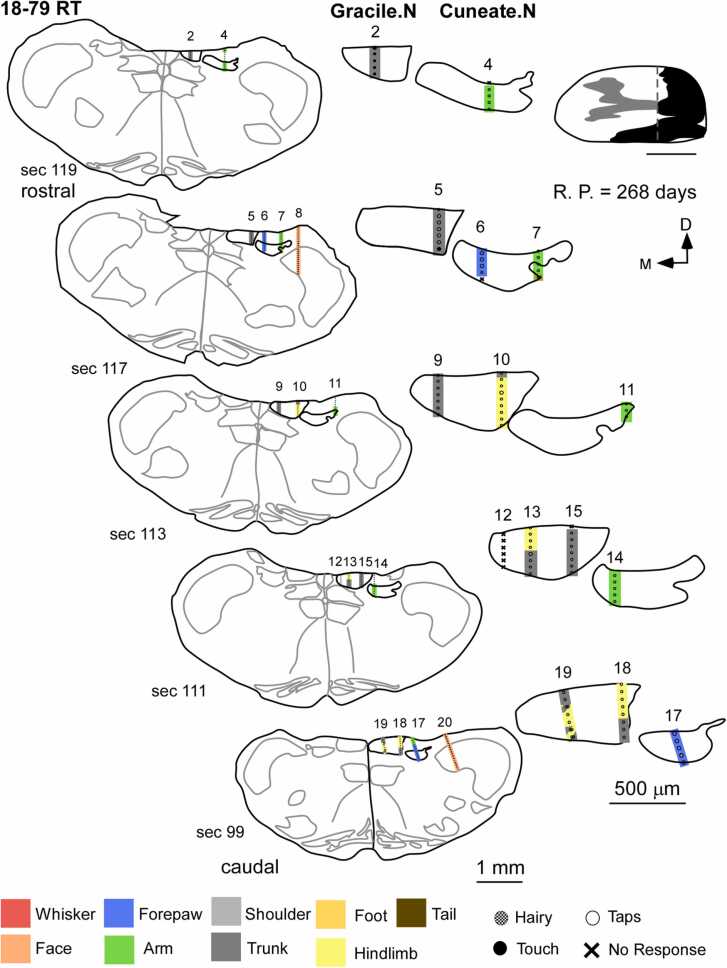
Organization of receptive fields in the medulla of a rat with a partial lesion of the dorsal columns. Schematics of coronal sections of the medulla (left) arranged along the rostro-caudal axis, displaying recording sites, following partial lesion of the dorsal columns (schematic in the top right corner). The right side shows the magnified schematics of the gracile and cuneate nuclei of the corresponding sections. The neurons in the gracile nucleus respond to stimulation of the hindlimb and trunk. The cuneate nucleus has receptive fields on the forepaw and forelimb. These responses are weak and mostly due to taps rather than light tactile stimuli. A few unresponsive sites are also observed. The receptive fields in the trigeminal nucleus were normal. There was no large-scale expansion of inputs from the face or any other intact body parts into the cuneate and gracile nucleus. Scale bar:1 mm (whole section schematic), 500 µm (inset); D, dorsal; M, medial; R. P., recovery period (post-lesion).

In five rats with partial dorsal column lesions, weak responses were observed in the cuneate and gracile nucleus. Responses were mostly evoked by hard taps on various parts of the body (Fig. 5). Neurons in the cuneate nucleus had receptive fields on the digits, pads of the forepaw, and arm. The gracile nucleus had receptive fields on the hindlimb, and trunk. Overall, most of the recording sites in the cuneate and gracile nuclei were responsive (Cuneate: 95.96 ± 2.2774 %; Gracile: 82.3868 ± 5.5885 %). Neurons in the trigeminal nucleus responded strongly to the stimulation of the face and demonstrated normal somatotopy. However, there was no expansion of chin or face inputs or representations of any other part of the body into the cuneate or gracile nucleus.

### 3.8 Analysis of responsive sites in the medulla of the normal and lesioned animals

**Fig. 6:**
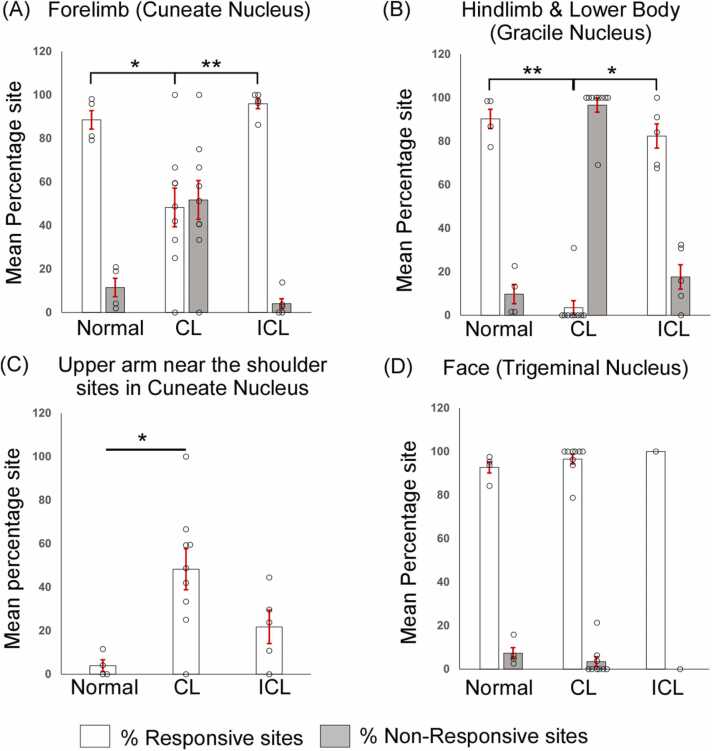
Comparison of recording sites in the dorsal column nuclei of medulla. Bar plots demonstrating mean ± SEM of percentage responsive and non-responsive sites in (A) cuneate nucleus and (B) gracile nucleus. The percentage of responsive sites was significantly less in both cuneate and gracile nuclei in animals with complete lesions compared to normal animals and those with incomplete lesions. (C) In the cuneate nucleus of complete lesioned animals, a significantly greater percentage of sites responding to the upper arm near the shoulder was observed compared to the normal animals. (D) There were no differences in the percentage of face-responsive sites across the groups in the trigeminal nucleus. The hollow circles shown in the plots represent data points from individual rats. CL, complete lesion; ICL, incomplete lesion. *, P ≤ 0.05; **, P ≤ 0.01; ***, P ≤ 0.001.

A comparison of the mean percentage of forelimb responsive sites across different groups demonstrated that there were significantly fewer responsive sites in animals with complete lesions compared to normal animals and animals with partial or incomplete dorsal column lesions (Fig. 6A; ANOVA, F_(2,15)_ = 9.893, P = 0.002; Holm-Sidak post hoc test, N vs CL, P = 0.013; CL vs ICL, P = 0.003). There were significantly fewer recording sites that were responsive to the hindlimb and lower body in rats with complete dorsal column lesions than in normal animals or animals with incomplete lesions (Fig. 6B; Kruskal-Wallis test, H = 14.175, P < 0.001; Dunn’s post hoc test, N vs CL, P = 0.007; CL vs ICL, P = 0.014). There was a significantly greater percentage of recording sites responsive to the upper arm near the shoulder in the cuneate nucleus of rats with complete dorsal column lesions compared to those in normal animals (ANOVA, F_(2,15)_ = 5.922, P = 0.013; N vs CL, P = 0.016; Fig. 6C). Responses from the face were not significantly different across normal animals and those with complete lesions (Fig. 6D), which demonstrates that even though trigeminal afferents were intact, they did not expand into the deafferented cuneate and gracile nucleus.

### 3.9 Relationship between organization of the somatosensory cortex and medulla: double mapping experiments

In order to determine the relationship between the organization of the cortex and the medulla after transection of the dorsal columns in adult rats, we used standard multiunit mapping procedures to map the cortex and the somatosensory nuclei of the medulla in the same animal. There was no expansion of inputs from the face and other body surfaces into the deafferented forelimb and hindlimb representations at the level of the SI or in the cuneate and gracile nucleus at the level of the medulla.

#### 3.9.1 Spared upper arm responses of the cuneate nucleus is observed in the SI in case of complete lesions

**Fig. 7:**
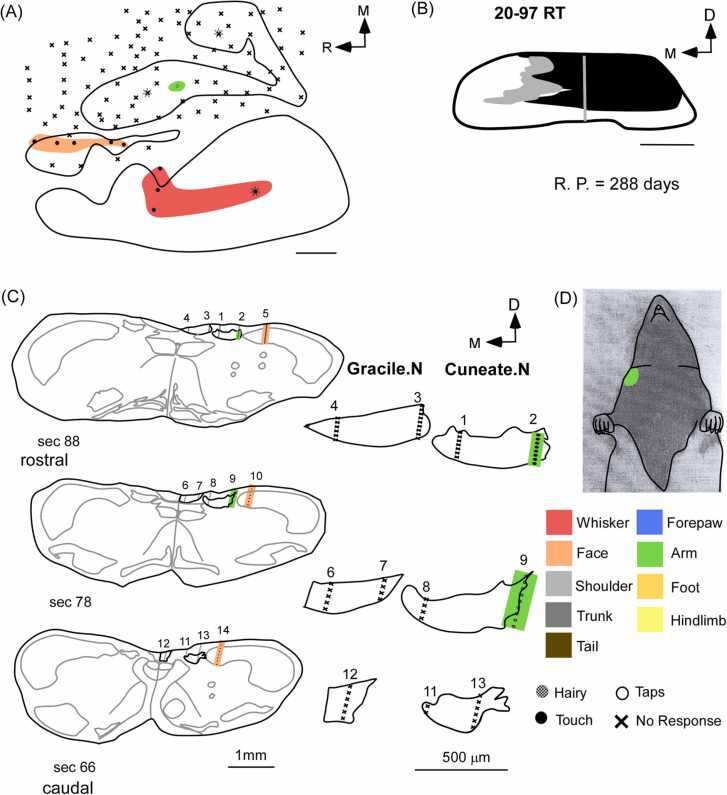
Relationship between receptive fields of the cortex and medulla in rats with complete lesions of dorsal columns. (A) The forelimb and hindlimb representations in the somatosensory cortex remain inactive except for a single site where a receptive field for the upper arm near the shoulder was observed. (B) The reconstruction of the spinal cord in the coronal plane demonstrates the extent of the lesion. (C) An electrophysiological map of the somatosensory nuclei of the medulla in the same animal shows that both the gracile and cuneate are largely inactive except for receptive fields on the upper arm near the shoulder in the cuneate nucleus. (D) The same receptive field as was seen in the cortex above was also encountered in the cuneate nucleus at the level of the medulla. No expansion of face or chin inputs was observed at the level of the medulla or the somatosensory cortex. Scale bar:1 mm (whole section schematic), 500 µm (inset). D, dorsal; M, medial; R, rostral; R. P., recovery period (post-lesion).

Out of the four animals that had undergone complete transections of the dorsal columns at the C3-C4 level, in two rats (20–97 RT and 17–54 RT), sites with receptive fields corresponding to the upper arm near the shoulder were encountered in the cortex and the medulla (Fig. 7A–D and Supplementary Fig. 6A). Electrophysiological mapping of the cortex of 20–97 RT and 17–54 RT revealed that the FBS, HBS, hindlimb, and trunk representations were completely inactive except for a single site in the arm representation that responded weakly to hard taps on the upper arm near the shoulder (receptive field shown in Fig. 7D). The lower jaw and whisker barrel cortex responded vigorously to the tactile stimulation of the face and whiskers, respectively. In the medulla of the same animal, we found that the gracile and cuneate nuclei largely stayed inactive. However, in some penetrations of the cuneate nucleus, recording sites were responsive to receptive fields on the upper arm near the shoulder. It is noteworthy to mention that this was the same receptive field observed in the cortex (Fig. 7C-D and Supplementary Fig. 6A). Furthermore, this was the only receptive field encountered across all responsive sites in the cuneate nucleus similar to our observations in the medulla mapping experiments described above.

In 20–96 RT, whereas the forelimb and hindlimb representation of the SI remained inactive, the face and whisker responses were observed at their expected locations. We also found that the gracile nucleus was inactive. However, in the cuneate nucleus, recording sites that were responsive to receptive fields on the upper arm near the shoulder were encountered. Responses were weak and evoked by hard taps (Supplementary Fig. 6B). The arm representation is a very narrow, diffused, and thin strip in the SI of rats, which is often covered by major blood vessels on the surface of the brain, making it very difficult to access during electrophysiological recordings. Furthermore, in rats with complete dorsal column lesions, only single sites responding to the upper arm near the shoulder were observed. Given the limitation of the technique and the sparse number of responsive sites, it is highly probable that we could not observe arm responses in the SI.

In 20–98 RT (Supplementary Fig. 6C), the forepaw, forelimb, foot, hindlimb, and trunk representations in the SI were found to be completely inactive and did not respond to stimulation of any other intact body part. In the same animal, the cuneate and gracile nuclei were found to be completely unresponsive probably due to the rostro-caudal level of the lesion (Supplementary Fig. 6D). There was no evidence of the large-scale expansion of the lower jaw or any other face inputs into the deafferented forelimb, hindlimb, and trunk representations either in the cortex or in the medulla.

#### 3.9.2 Partial lesions result in spared responses and demonstrate similar receptive fields at both levels

**Fig. 8:**
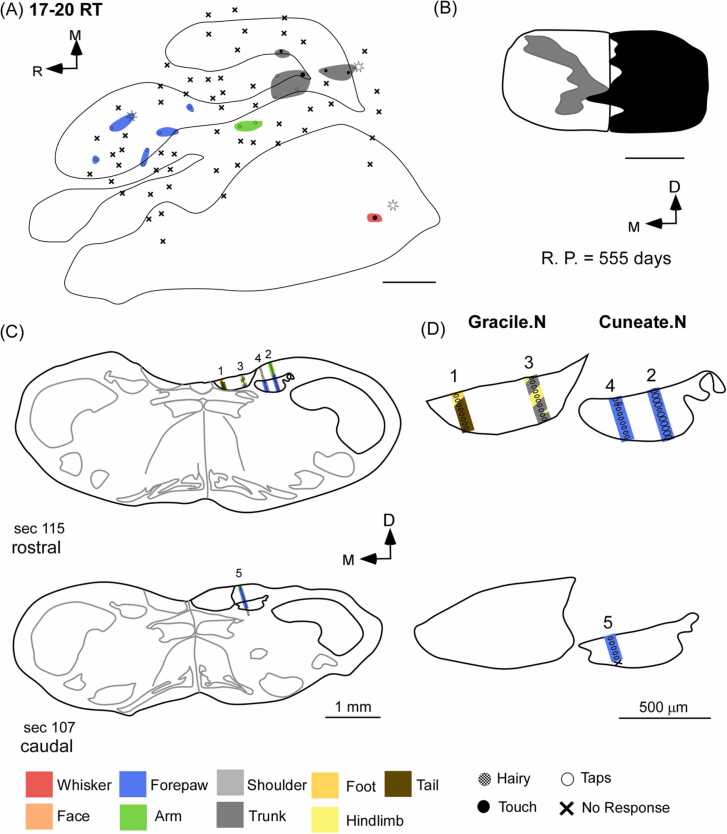
Relationship between receptive fields of the cortex and medulla in a rat with partial lesion of the dorsal columns. (A) The electrophysiological map of the somatosensory cortex of a rat with a partial dorsal column lesion appears as a mosaic of responsive and unresponsive sites. Responses to the forepaw, arm, and trunk were observed at their expected locations in the cortex. (B) The coronal reconstruction of the spinal cord demonstrates the extent of the lesion. (C and D) The sites of electrode penetration are shown in the schematics of coronal sections of the medulla on the left and magnified views of the corresponding gracile and cuneate nuclei are shown on the right. Receptive fields of the trunk, hindlimb, and tail were observed in the gracile nucleus and that of the forepaw was found in the cuneate nucleus. Large-scale expansion of inputs of the face or any other intact body parts was not observed in the cortex or medulla. Scale bar:1 mm (whole section schematic), 500 µm (inset). D, dorsal; M, medial; R, rostral; R. P., recovery period (post-lesion).

In rats (n = 3) that had undergone partial lesions of the dorsal columns, there was an absence of large-scale expansion of face inputs into the forelimb or the hindlimb representations both at the level of the cortex and at the level of the medulla. Due to the sparing of dorsal column fibers, weak responses were evoked by taps on various body parts, such as the forepaw, arm, and trunk representations at the level of the cortex (Fig. 8A-B). Similar receptive fields were observed in the gracile and cuneate nuclei at the level of the medulla (Fig. 8C-D).

#### 3.9.3 Relationship between spared responses in SI and medulla

**Fig. 9:**
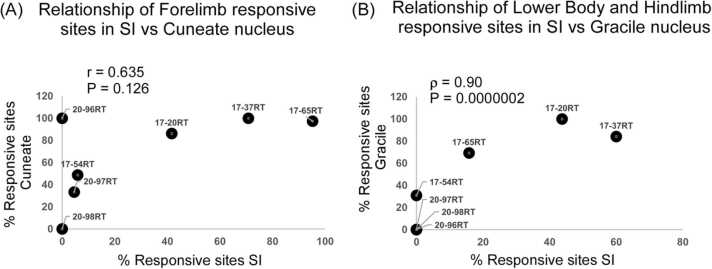
Correlation analysis between SI and dorsal column nuclei of medulla in lesioned rats. (A) The percentage of forelimb-responsive sites from the SI and the cuneate nucleus were not significantly correlated, possibly due to the higher occurrence of recording sites responding to the upper arm near the shoulder in the cuneate nucleus compared to those in the SI. (B) A positive correlation was observed in the hindlimb and lower body responses of SI and the gracile nucleus of the medulla.

A correlation analysis between spared percentage responsive sites demonstrated that there was no significant correlation between the forelimb responses in the cuneate nucleus and the SI (Pearson product-moment correlation, r = 0.635, P = 0.126; Fig. 9A). This was due to the higher number of recording sites responsive to the spared upper arm near the shoulder in the cuneate nucleus compared to SI. A positive correlation was observed for the hindlimb and the lower body responsive sites between the gracile nucleus and the SI (Spearman rank order correlation, ρ = 0.900, P = 0.0000002; Fig. 9B).

## 4 Discussion

We studied the effects of chronic dorsal column lesions in adult rats at the level of the cortex and the medulla. We found that after a prolonged recovery period (3 weeks – 18 months) following transection of the dorsal columns at the cervical C3-C4 level, the deafferented forepaw, hindlimb and trunk representations in SI remained inactive and there was no expansion of face inputs into these regions. The inputs from face that were spared and their respective neural responses were found at their expected locations in SI, as ascertained by CO isomorph on histological sections. At a few recording sites, responses to spared upper arm near the shoulder were observed. Similarly, it was found that the gracile and the cuneate nucleus remained largely inactive except for a few sites in the cuneate nucleus that were responsive to the upper arm near the shoulder. Inputs from the upper arm near the shoulder were spared in spite of a complete transection of the dorsal columns along the mediolateral plane as they enter the spinal cord rostral to the site of the lesion (C3-C4), as observed in similar studies (Jain et al., 2003, Takahashi and Nakajima, 1996). There was no expansion of face inputs or afferents from the adjacent trigeminal nucleus into the deafferented cuneate nucleus at the level of the medulla.

### 4.1 Reorganization of the primary somatosensory cortex

Earlier anatomical studies have investigated the effects of peripheral perturbations on the central organization. For example, neonatal forelimb removal in rats disrupted the anatomical representation of the forepaw (FBS) and led to the expansion of the hind paw representation in SI (Dawson and Killackey, 1987, Killackey and Dawson, 1989). Previous studies in rats have shown that the amputation of a forelimb at early postnatal stages or in adulthood results in the expansion of inputs from the stump into the FBS which normally responds to the digits and pads of the forepaw (Lane et al., 1995, Pearson et al., 2003, Pearson et al., 1999). These studies also reported that the amputation caused a complete disruption of CO-dense, barrel-like structures in the FBS, whereas, in adult rats, the FBS and HBS were discernible. Similarly, dorsal column lesions at the C3-C4 cervical level in postnatal day 3 rat pups resulted in very faint CO-dense barrels or their complete absence in the FBS and HBS of SI (Jain et al., 2003). Spared responses from the upper arm that enter the spinal cord rostral to the C3-C4 lesion site activated parts of the deafferented forepaw cortex to varying degrees, demonstrating the expansion of the upper arm inputs into the forepaw cortex. In comparison, the head or neck inputs expanded to a very limited extent into the deafferented forepaw cortex. Taken together, these findings suggest that the developing rat somatosensory system responds very differently to spared afferents from the forelimb and face (Jain et al., 2003).

However, when dorsal column lesions were performed at thoracic T6-T8 levels in adult rats, it was found that the CO modules of the HBS were not disrupted and could be easily identified (Jain et al., 1995). In the present study, deafferentation of the forelimb and the hindlimb by transection of dorsal columns at C3-C4 levels of adult rats did not disrupt the CO-dense barrel or module-like patterns of the FBS and HBS. Even though the effects of dorsal column lesions and forelimb amputation on the CO isomorph is quite similar, the neurophysiological maps obtained after the spinal cord lesions and amputation differ in terms of the extent of reactivation or expansion of intact inputs into deafferented territories of SI. Pearson et al. have reported an expansion of the shoulder representation in the deafferented FBS after forelimb amputation in adult rats (Pearson et al., 2003, Pearson et al., 2001). Similarly, if the sciatic nerve was transected in adult rats, saphenous nerve inputs invade the territories of the hindlimb cortex which is normally innervated by the sciatic nerve (Wall and Cusick, 1984). However, dorsal column lesions at thoracic levels in adult rats did not reactivate the hind paw cortex or result in the expansion of the intact forelimb or face representations into the deafferented hind paw cortex (Jain et al., 1995). Similarly, in the present study, the deafferented forepaw and hind paw cortex stayed inactive even after prolonged recovery periods such as 18 months after dorsal column lesions and there was no expansion of spared shoulder inputs into the FBS.

The somatosensory cortex of adult primates is capable of reorganization in response to different types of peripheral and central deafferentations. However, the reorganization differs in terms of its limits depending upon the survival times and extent of deafferentations. For example, dorsal column lesions were performed at mid-cervical levels (C4-C5) which deafferents the D2–5 digits while sparing the D1 digit to explore the extent of cortical plasticity in area 3b and area 1 of adult squirrel monkeys (Qi et al., 2011). The authors reported abnormal topography, weak responses, a high incidence of unresponsive sites, and receptive fields on the dorsal surface of the digits. There was no expansion of face inputs in the deprived hand area of 3b. Experiments involving transections of median and/or ulnar nerves have revealed an expansion of radial nerve territories in the deprived median and ulnar nerve cortical representations in the hand representation of area 3b in adult primates (Garraghty and Kaas, 1991a, Kolarik et al., 1994, Merzenich et al., 1983). Acute studies on nerve transections in adult primates reported very minimal or no face expansion in the deafferented hand representation of area 3b (Kolarik et al., 1994, Silva et al., 1996). However, chronic and massive deafferentations like dorsal rhizotomies (C2-T4) or cervical dorsal column lesions in adult primates result in large-scale reorganization characterized by the expansion of face inputs into the deafferented hand representation (Halder et al., 2018, Jain et al., 2008, Jain et al., 2000, Jain et al., 1997, Kambi et al., 2014, Pons, 1994). Hence, immediate changes in the somatotopy that occur after small peripheral perturbations differ from chronic long-term changes following massive deafferentations in adult primates.

### 4.2 Reorganization at the level of the medulla

Killackey and Dawson observed that prenatal forelimb removal disrupts the FBS and led to expansion of the hindlimb representation at the level of SI and at the level of the brainstem (Killackey and Dawson, 1989). Rhoades and co-workers demonstrated expansion of afferents from the gracile nucleus into the denervated cuneate nucleus by employing electrophysiology and anatomical tracers in rats that had undergone fetal forelimb amputation (Rhoades et al., 1993). In another study, neonatal forelimb removal resulted in a similar expansion of sciatic nerve projections into the cuneate nucleus which showed that neurons had receptive fields on both the stump and hindlimb (Lane et al., 1995). It has been observed that forelimb amputation in adult rats results in the expansion of the trunk and other intact body parts in the non-cluster zones (the region outside the CO-dense barrel region of the cuneate nucleus) while the central CO-dense barrel region was largely unresponsive to tactile stimulation of any part of the body (Li et al., 2013). These studies demonstrate plasticity at the level of the medulla in response to peripheral manipulations that occurred during or before the complete development of the somatosensory system of rats. In the present study, we did not find any expansion of the intact trigeminal nucleus into the deafferented cuneate nucleus, following C3-C4 cervical lesions in adult rats. This shows that the somatosensory system of adult rats is somewhat limited in its capability to reorganize, compared to that of a developing animal.

Manipulations of the periphery lead to somatotopic reorganizations of the dorsal column nuclei in the medulla of primates. Xu and Wall have demonstrated that within minutes to hours of paired median and ulnar nerve transections, neurons in the cuneate nucleus that normally respond to tactile stimulation of the glabrous ventral surface of the hand started responding to stimulation of its hairy dorsal surface. There were more unresponsive sites than observed in a normal animal. It is noteworthy that there was no expansion of face inputs into the cuneate nucleus after median and ulnar nerve transections in these acute studies (Xu and Wall, 1999a, Xu and Wall, 1999b). However, long-standing deafferentations like dorsal column lesions at high cervical levels of adult primates result in the large-scale reorganization, characterized by an expansion of the intact face inputs into the deafferented cuneate nucleus (Halder et al., 2018, Kambi et al., 2014). Tracer studies in adult primates have demonstrated the growth of afferents from the trigeminal nucleus into the cuneate nucleus of adult primates after cervical dorsal column lesions or therapeutic amputation of the arm (Jain et al., 2000). Furthermore, an expansion of the arm, upper shoulder, neck, and occiput, in addition to the ipsilateral chin responses occur in the medial part of the cuneate nucleus and pars rotunda, which mostly respond to stimulation of hands in normal monkeys (Halder et al., 2018). Therefore, our results in adult rats are similar to those in primates because we also observed responses to stimulation of the upper arm near the shoulder and a higher occurrence of unresponsive sites following C3-C4 dorsal column lesions. However, our results differ from primates since we did not find any responses from the chin, lower face, or inputs from the head within the cuneate nucleus of rats.

### 4.3 Possible mechanisms underlying reorganization

The discovery of the massive expansion of the face representation into the deafferented hand representation of the SI of adult monkeys following dorsal rhizotomies C2-T5 ushered in an era of exploration of injury-induced neuroplasticity in the adult brain (Pons et al., 1991). Previous studies had shown that peripheral manipulations lead to central reorganizations at the levels of the somatosensory system, that is, the spinal cord, medulla, thalamus, and cortex of adult animals (Merzenich and Kaas, 1982). The SI and VPL nucleus of the thalamus are known to reorganize in cases of amputations, peripheral nerve injuries, dorsal column lesions, and dorsal rhizotomy in rats and monkeys (Florence et al., 2000, Garraghty and Kaas, 1991b, Jain et al., 2008, Jones and Pons, 1998, Li et al., 2014). It is proposed that the immediate reorganization that follows common deafferentation procedures, for example, nerve transections and digit denervation, are mostly due to the unmasking or potentiation of existing connections by disinhibition (Byrne and Calford, 1991, Calford and Tweedale, 1991, Churchill et al., 1998, Silva et al., 1996, Xu and Wall, 1999b). Electrophysiological recordings and tracer studies in both rats and monkeys have proved that the reorganization observed in the cortex is not due to intracortical connections (Chand and Jain, 2015, Kambi et al., 2014, Pearson et al., 2001). Pons proposed that the brainstem was the seat of large-scale reorganization marked by face expansion observed at the level of the cortex (Pons et al., 1991). A number of tracer studies have demonstrated that the sprouting of afferents from intact or spared representations into the deafferented zones of the cuneate nucleus in adult animals is the mechanism underlying the reorganization following long-term deprivation of somatosensory inputs by nerve section, dorsal column lesions, amputation and dorsal rhizotomies (Florence et al., 1994, Jain et al., 2000, Rhoades et al., 1993, Sengelaub et al., 1997, Wu and Kaas, 2002). Interestingly, Qi et al. reported a lack of face expansion in area 3b of adult squirrel monkeys 7–9 weeks after dorsal column lesions, which might not be enough time for the growth of new connections from the trigeminal to the cuneate nucleus (Qi et al., 2011). The sprouting of afferents from the trigeminal nucleus to the deafferented cuneate nucleus following dorsal column lesions or amputations has been reported after 18 months of recovery or more in primates (Jain et al., 2000). Moreover, numerous electrophysiological studies have demonstrated and compared the contribution of injury-induced reorganization of the cuneate nucleus to cortical plasticity (Churchill et al., 2001, Florence and Kaas, 1995, Kambi et al., 2014, Lane et al., 1995, Xu and Wall, 1999b). While most of the earlier studies on the relationship between reorganization in the brainstem and in the SI were correlational, the study by Kambi et al. (Kambi et al., 2014) has demonstrated the causal relationship between the expansion of face inputs into the deafferented hand representation of area 3b in monkeys and the cuneate nucleus. This study has established that the cuneate nucleus is the driver of cortical reorganization and has ruled out VP (thalamus)-mediated plasticity. The two key factors that affect the extent of injury-induced reorganization, are the extent of the deafferentations or injury and the duration of the recovery period. In the present study, we employed higher cervical (C3-C4) dorsal column lesions that causes massive deafferentations and obtained physiological maps after a prolonged recovery period (3 weeks - 18 months). In spite of the above, the expansion of the face or other intact body parts was not observed in the SI and cuneate nucleus of adult rats. Due to the high fidelity of connections among isorepresentations throughout the somatosensory axis, it is unlikely that such large-scale expansions would be observed at the level of the thalamus when it is absent in the medulla from which it receives ascending somatosensory inputs. Furthermore, large-scale expansions are also absent in SI, which receives projections from VP.

A lesion at the upper cervical (C3-C4) level interrupts somatosensory inputs from the trunk, tail, hindlimb, and forelimb. However, somatosensory inputs from the upper arm near the shoulder which enter the spinal cord rostral to the site of the lesion are spared (Jain et al., 2003, Takahashi and Nakajima, 1996). Hence, in the present study, unlike the gracile nucleus and the hindlimb and trunk representation of SI, the cuneate nucleus and forelimb representation of SI are not completely deafferented even after complete transection of the dorsal columns along the mediolateral plane of the spinal cord. Therefore, the hindlimb and trunk cortex in SI and the gracile nucleus in the medulla remain completely inactive, which is reflected in the correlation analysis (Fig. 9B).

A greater number of sites responding to the upper arm near the shoulder in the cuneate nucleus were observed compared to that at the level of the SI which resulted in a lack of significant correlation between the cuneate nucleus and SI (Fig. 9A). These findings may have resulted from the fact that somatosensory inputs are not only relayed but also transformed or suppressed as they ascend from the somatosensory nuclei of the medulla to the VPL, VPM of the thalamus and subsequently to SI (Halder et al., 2018, Lane et al., 1997, Lane et al., 1995). The topographic organization within the somatosensory nuclei and receptive fields of neurons at each level are dynamically maintained by the ascending inputs as well as the numerous feedforward, feedback, and lateral connections which also include inhibitory inputs.

Another study has shown that neonatal forelimb amputation resulted in the expansion of sciatic nerve afferents into the cuneate nucleus. Neurons in the cuneate nucleus had acquired receptive fields on the forelimb stump and the hindlimb. Similar to our results, the authors also reported significantly fewer recording sites responding to both forelimb stump and hindlimb stimulation at the SI compared to the cuneate nucleus of the medulla (Lane et al., 1995). Furthermore, in subsequent studies, it was demonstrated that this is due to suppression at the thalamic or cortical level which is mediated by GABA (Lane et al., 1997, Lane et al., 1995). Since the arm representation in SI of rats is very narrow and often occluded by major surface vasculature, it is less accessible for electrophysiological recording. It is therefore possible that we observed fewer sites responding to the upper arm near the shoulder in the SI due to this technical limitation.

Massive deafferentations like spinal cord injury or amputation of limbs lead to the reorganization of receptive fields and alter the somatotopic maps at all levels of the somatosensory axis (Wall et al., 2002). Subcortical nuclei such as the cuneate nucleus in the medulla which are located in close proximity to the site of injury might undergo rapid and extensive reorganization. Suppression mechanisms in the cortex and the thalamus (Halder et al., 2018, Lane et al., 1997) might provide protection from such sudden and drastic large-scale subcortical reorganizations, which might otherwise prove detrimental to the survival of the animal (Wall et al., 2002).

### 4.4 Relations to motor reorganizations

Brains of mammals like primates and rodents share the general plan of organization of the sensory-motor pathways. Despite these superficial similarities, there are intricate and unique inter-species differences that actually reflect evolutionary specializations that have enabled each species to survive and adapt perfectly to their particular ecological niche and habitat.

The organization of the motor systems between primates and rodents differs considerably (Badi et al., 2021, Sinopoulou et al., 2022). It is well established that the corticospinal tract descends at the base of the dorsal columns in rats (Tracey, 2004). Sensory-motor integration is crucial for both primates and rodents to survive and quickly respond to the rapid flux of stimuli arising from the transient and ever-changing surrounding environment. However, it is achieved using very different strategies in the two species. For example, the remarkable dexterity observed in hand movements of primates like rhesus macaques can be attributed to the narrow and heavy focus of the corticospinal projectome on the motor outputs, which reflects the segregation of sensory and motor systems. In stark contrast, the corticospinal projectome of rats, which is related to hand control, emphasizes sensory-motor integration by sending efference copies through the majority of axon collaterals to sensory systems, in order to achieve modulation of motor movements (Sinopoulou et al., 2022).

Such fundamental inter-species differences between the brain of primates and rodents during normal conditions form the basis of very different outcomes in case of injury-induced reorganizations in the two species. For example, rats appear to be much more robust in response to motor cortical injury due to the redundancy of motor output pathways, when compared to primates (Sinopoulou et al., 2022). On the other hand, sprouting in the spinal cord after spinal cord injury facilitates more pronounced spontaneous functional motor recovery in primates than in rodents (Friedli et al., 2015, Sinopoulou et al., 2022). The present study shows that the lack of large-scale face expansion in the somatosensory system of adult rats, which is surprising since unilateral corticospinal tract lesions at cervical levels lead to a complete reorganization marked by the expansion of neighboring zones like whiskers and neck into the de-efferented forelimb motor cortex of rats (Tandon et al., 2013).

However, sprouting might not always be advantageous. Chronic lesion of dorsal columns in primates results in spontaneous sprouting of intact trigeminal inputs into the deafferented cuneate nucleus in the medulla that might lead to pathological conditions like phantom sensations (Jain et al., 2000, Kambi et al., 2014, Moore et al., 2000). Unlike in primates, our data demonstrate that the somatosensory system of rats does not exhibit such large-scale face expansion at the level of the SI and the medulla even after prolonged recovery period. This lack of reorganization might actually aid in the survival of the animal by shielding it from sudden drastic alterations in the perception of body surface following massive deafferentations like cervical spinal cord injury. Similarly, in motor systems of primates, the loss of corticorubral projections from the premotor cortex (PM) and primary motor cortex (M1) in response to cervical spinal cord injury might contribute to functional recovery (Borgognon and Rouiller, 2023). However, in rats, forced use of impaired limb following stroke, cause axonal sprouting of the motor corticorubral pathway which in turn leads to functional recovery (Borgognon and Rouiller, 2023, Ishida et al., 2016).

Hence, a thorough understanding of neurophysiological differences between the species of model systems like primates and rodents is the first step towards the development of somatosensory neuroprosthetics (SNP) which mimics real tactile stimuli and provides somatosensory feedback at appropriate sensory areas across the neural axis in human patients who have suffered spinal cord injuries (Badi et al., 2021).

## 5 Conclusion

In the large body of literature that has investigated lesion-induced brain reorganization, it is established that the species of the animal, the type of injury and the age of the animal are key factors in the nature and extent of reorganization (Bowlus et al., 2003, McKinley and Smith, 1990). The results obtained from our experiments can be appreciated for their true merit only when compared with and contrasted against the effects of dorsal column lesions on the primate somatosensory system. At a first glance, our data shows that the deafferented representations of the forelimb, hindlimb, and trunk in SI and medulla remain inactive in adult rats unlike that in primates where the reorganization after dorsal column lesions is marked by a clear expansion of face and chin inputs into the deafferented hand area of 3b and the cuneate nucleus. The large-scale face expansion in area 3b of primates is driven by reorganization in the cuneate nucleus, which is mediated by the sprouting of intact trigeminal afferents into the adjacent cuneate nucleus (Jain et al., 2000, Kambi et al., 2014). However, the sprouting and growth of new connections at the level of the brainstem require a prolonged period of time (Qi et al., 2011). Hence, the acute studies in primates that explored the immediate effects of dorsal column lesions or other kinds of deafferentations like denervation or amputation did not report an expansion of intact chin or face inputs into the deafferented hand representation in area 3b (Kolarik et al., 1994, Qi et al., 2011, Silva et al., 1996). The large zones of inactive recording sites in somatotopic maps of the SI of adult rats obtained after complete dorsal column lesions in our data resemble that of the acute somatotopic maps of the cortex of squirrel monkeys obtained hours after complete lesions of the dorsal columns, which is largely inactive and devoid of face expansion (Jain et al., 1997). Our results in the cuneate nucleus of adult rats demonstrate an occurrence of recording sites that are responsive to stimulation of spared upper arm near the shoulder similar to observations in adult primates after cervical dorsal column lesions (Halder et al., 2018). Our results therefore suggest that the capability of reorganization of the adult rat somatosensory system following dorsal column lesions is severely limited. Hence, the somatosensory system of adult rats and primates respond differently to dorsal column lesions. This difference might be further explored to discover novel therapeutic methods to restrict maladaptive reorganizations that lead to phantom sensations.

## Ethics statement

The procedures performed on all animals used in this study were approved by the Institutional Animal Ethics Committee (IAEC) of the National Brain Research Centre, and the Committee for the Purpose of Control and Supervision of Experiment in Animals (CPCSEA), Government of India.

## Funding information

The study is supported by National Brain Research Centre Core Funds, Manesar, India. The sponsor did not have any role in study design.

## CRediT authorship contribution statement

**Atanu Datta:** Conceptualization, Data curation; Formal analysis, Investigation, Methodology, Project administration, Software, Validation, Visualization, Writing – original draft, Writing – review & editing.

## Declaration of Competing Interest

The author declares no conflict of interest.

## Data Availability

The data presented in the study are included in the article, further inquiries can be directed to the corresponding author.
