# Supplementary material for: The effect of dorsal column lesions in the primary somatosensory cortex and medulla of adult rats
Source: IBRO Neurosci Rep. 2023 May 14;14:466–82. doi: 10.1016/j.ibneur.2023.05.005 (PMC10238474; doi:10.1016/j.ibneur.2023.05.005)
Supplement: Supplementary file 1 — Supplementary material [file mmc1.docx]

Supplementary Material

**The Effect of Dorsal Column Lesions in the Primary Somatosensory Cortex and Medulla of Adult Rats**

Atanu Datta*^1^

^1^National Brain Research Centre, Manesar, Gurugram, Haryana-122052, India

*** Correspondence:** Atanu Datta, email: [atanudatta1989@gmail.com](mailto:atanudatta1989@gmail.com)

**Supplementary Figure Legend**

**Supplementary Fig. 1.** Methods employed in the neurophysiological mapping of the primary somatosensory cortex (SI).

(**A**) The figure shows an adult rat's brain and the spinal cord injury that targeted the dorsal columns on the right. The inset shows the site of the lesion (cervical C3-C4) marked by the presence of scar tissue (*red arrowhead*). Scale bar: 1cm, 1mm. (**B**) Dark-field photomicrograph of a horizontal section of the rat spinal cord demonstrating a lesion of the dorsal columns (rectangle); dotted line denotes the midline and red arrowhead denotes the lesion. A high-magnification view of the lesion site shows that it has crossed over the midline and completely ruptured the dorsal columns (inset). The reconstruction of the spinal cord (below) in the coronal plane using camera lucida tracings of the horizontal sections provides an estimate of the extent of the dorsal column lesion. Scale bar: 1mm. (**C**) A photograph of the brain of an anesthetized adult rat after a craniotomy was performed, using the bregma (*black arrowhead*) as a landmark to access the entire extent of the SI for electrophysiological recordings. (**D**) A photomicrograph of a section of the flattened cortex, stained for cytochrome oxidase, demonstrates the CO isomorph with its dense barrel-like patterns. Scale bar: 1mm. (**E**) Photograph of the flattened cortex after the brain was removed following transcardial perfusion of the rat with phosphate-buffered saline and paraformaldehyde. Scale bar: 5mm. D, dorsal; M, medial; R, rostral.

**Supplementary Fig. 2**. Methods employed for mapping the dorsal column nuclei of the medulla.

**(A**) A photograph of the surface of the medulla demonstrating the region from which recordings were performed near the obex. The *black arrowheads* indicate the position of the obex and the dotted line shows the overhanging cerebellum beneath the occipital bone. (**B**) Photomicrographs of coronal sections of the medulla stained for cytochrome oxidase. *Black arrowheads* indicate the site of electrolytic microlesions placed after the commencement of the recording sessions. (**C**) A magnified image of the cuneate nucleus of section 123 (dotted box) demonstrating the presence of the central CO-dense cluster of barrelettes. (**D**) A coronal section of the medulla demonstrates the different types of electrolytic lesions. *Black arrowheads* show point lesions at specific depths, and continuous lesions help to identify the angle of electrode penetration. (**E**) A magnified photomicrograph of the cuneate nucleus showing the electrode track passing through the barrelettes of the cluster zone of the cuneate nucleus. Scale bar: 1mm and 500 µm (inset); C, cuneate nucleus; D, dorsal; G, gracile nucleus; M, medial; R, rostral; T, trigeminal nucleus.

**Supplementary Fig. 3.** Example of responsive and unresponsive sites in SI.

(**A**) The reconstructed neurophysiological map of SI of a normal rat 16-04 RT. The numbers in the physiological map and the schematics of body parts denote the recording sites and their corresponding receptive fields respectively. (**B**) A drawing of the ventral forepaw demonstrating the digits (D1-D5) and pads. The figures to the right depict the receptive fields (*blue patches*) of the corresponding recording sites. (**C**) Examples of arm receptive fields of the recording sites in the arm cortex. (**D**) A drawing of the ventral hind paw demonstrating the digits (T1-T5) and pads. The figures to the right depict the hind paw receptive fields (*yellow patches*) of the corresponding recording sites. (**E**) The schematic of the ventral face depicts receptive fields on the lower jaw of the rat. (**F**) The neurophysiological map of SI was obtained from a rat (20-98 RT) with a complete dorsal column lesion (as shown in the spinal cord reconstruction below). The forelimb, hindlimb, and lower body representation were completely inactive and are marked with crosses. Each cross represents a recording site that was unresponsive to tactile stimulation of any parts of the body. (**G**) Examples of receptive fields in the lower jaw representations which demonstrated that face inputs were intact. Scale bar: 1mm. M, medial; R, rostral.

**Supplementary Fig. 4.** The reconstructed neurophysiological map of SI of a rat with complete dorsal column lesion.

The electrophysiological mapping of the SI of 16-109 RT, which had undergone a complete transection of dorsal columns as demonstrated by the spinal cord reconstruction, revealed a few responsive sites that responded weakly to stimulation of the forepaw and the proximal tail at their usual locations possibly due to a few spared fibers. Scale Bar: 1mm. D, dorsal; M, medial; R, rostral; R. P., recovery period (post-lesion).

**Supplementary Fig. 5.** Example of responsive and unresponsive sites in the medulla.

(**A**) A schematic of the coronal section (section 88) of the medulla of a normal rat (20-103 RT) shows the sites of electrode penetrations in the somatosensory nuclei of the medulla. (**B**) A magnified view of these electrode penetrations in the cuneate nucleus. Examples of recording sites in the electrode penetrations are depicted along with their corresponding receptive fields denoted by the colored patches (in *blue*) on the drawings of the ventral forepaw. The letters denote the recording sites. (**C**) Similarly, the recording sites in the gracile nucleus along with their corresponding receptive fields are shown in *yellow*. (**D**) The recording sites in the trigeminal nucleus and their respective receptive fields are shown on the schematics of the face of the normal rat. (**E**) A schematic of the coronal section (section 130) of the medulla of a rat (20-101 RT) with a complete dorsal column lesion (coronal reconstruction of lesion site shown below) demonstrates the sites of electrode penetrations in the somatosensory nuclei of the medulla. (**F**) A magnified view of these electrode penetrations in the gracile and cuneate nucleus. Examples of unresponsive recording sites are shown in the enlarged schematic of the gracile nucleus. The cuneate nucleus appears as a mosaic of responsive and unresponsive sites. All the responsive recording sites had receptive fields on the upper arm near the shoulder which is depicted on the drawing of the rat by a colored patch in *green*. The letters denote the recording sites. (**G**) The recording sites in the trigeminal nucleus and the respective receptive fields are shown on the schematics of the face of the rat with dorsal column lesions. Scale bar for section schematics: 1mm; Scale bar for the magnified nuclei: 500 µm. D, dorsal; M, medial.

**Supplementary Fig. 6.** Organization of the receptive fields in double mapping experiments in rats with complete dorsal column lesions.

(**A**) The SI remained mostly inactive after the lesion in 17-54 RT (as shown in the spinal cord lesion reconstruction) except for one site that responded weakly to hard taps on the upper arm near the shoulder. However, the face and whisker inputs stayed intact. A similar receptive field was observed at the level of the medulla in the cuneate. A few weak responses were elicited by stimulation of the foot, hindlimb, and trunk in the gracile nucleus. Both nuclei displayed a mosaic of responsive and unresponsive sites with no expansion of intact body parts as observed in the cortex. (**B**) Similarly, in 20-96 RT, the SI largely stayed inactive but facial inputs were intact and at the level of the medulla, responses to taps on the upper arm near the shoulder were observed in the cuneate. In this case, the reconstruction of the spinal cord demonstrated the complete obliteration of the dorsal columns. (**C**) In 20-98 RT, after the dorsal column lesion, as demonstrated by the spinal cord reconstruction, the forelimb, and the hindlimb cortex stayed inactive. At the level of the medulla both the nuclei, that is, gracile and cuneate did not show any responsive sites. (**D**) A photograph of the spinal cord of 20-98 RT demonstrating the presence of scar tissue at the rostro-caudal level (C2-C4 level). Scale bar: 1mm. R. P., recovery period (post-lesion).
