# Supplementary figures and images for: The effect of dorsal column lesions in the primary somatosensory cortex and medulla of adult rats
Source: IBRO Neurosci Rep. 2023 May 14;14:466–82. doi: 10.1016/j.ibneur.2023.05.005 (PMC10238474; doi:10.1016/j.ibneur.2023.05.005)

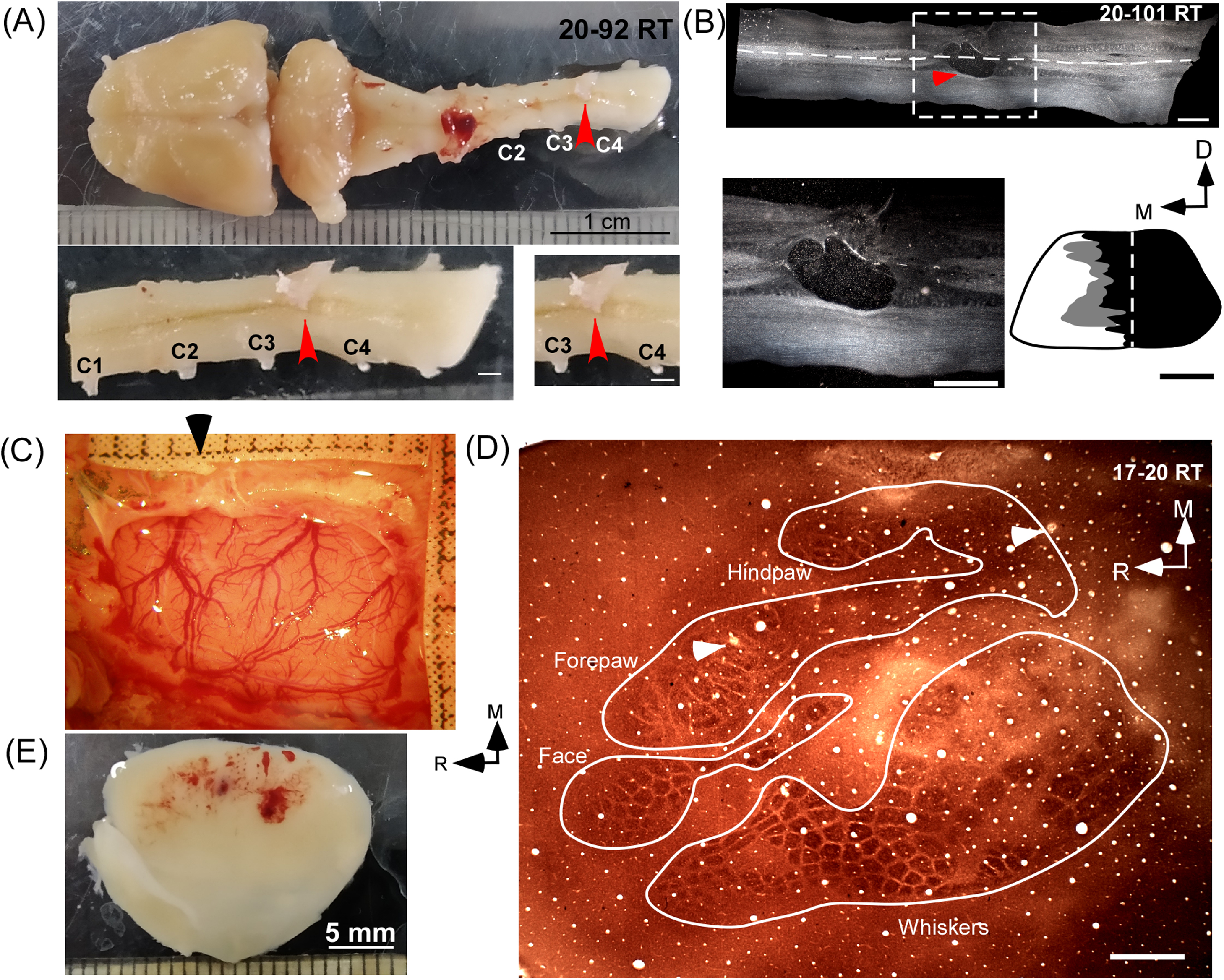

Supplement: Supplementary file 2 — Supplementary material [file mmc2.jpg]

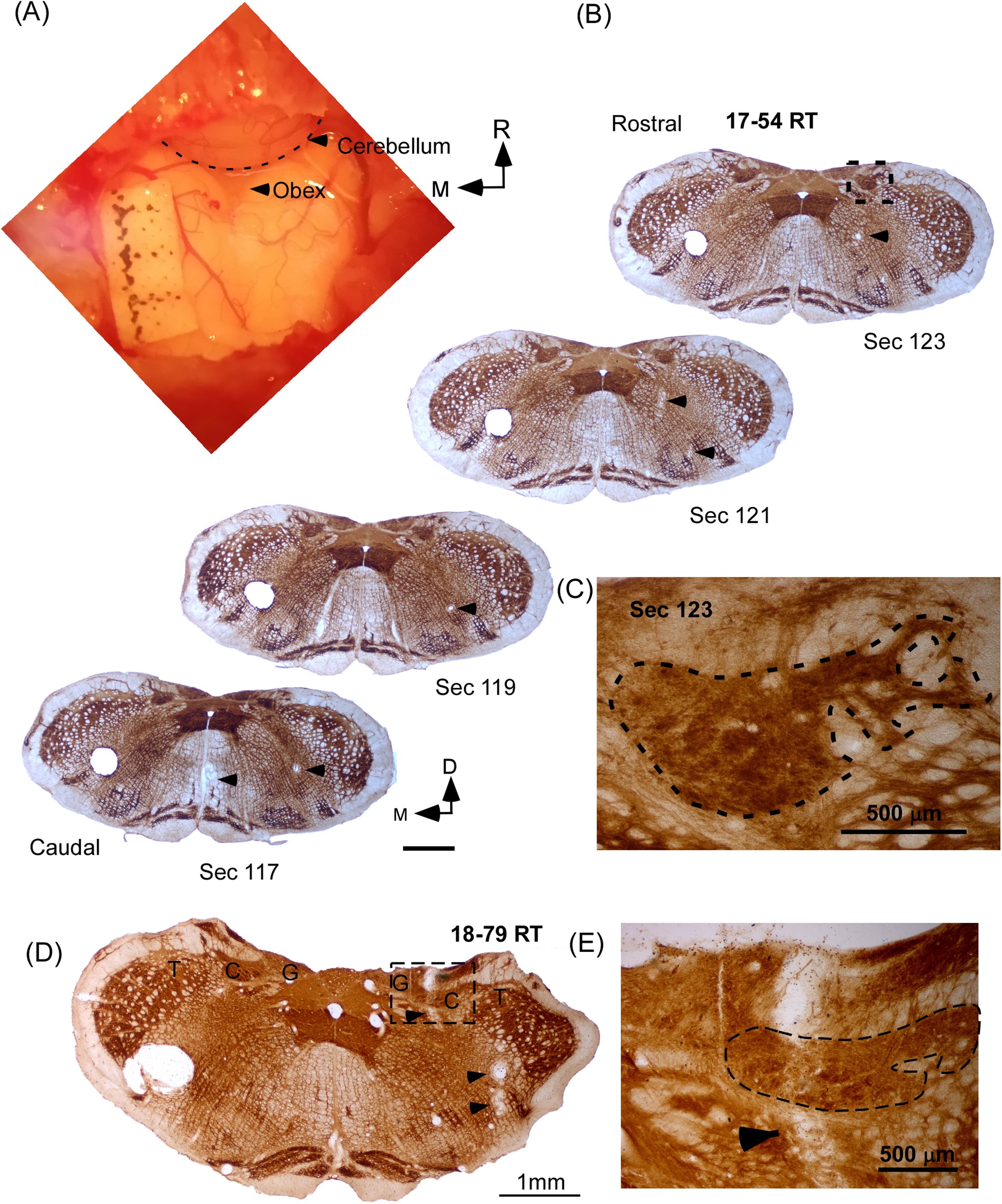

Supplement: Supplementary file 3 — Supplementary material [file mmc3.jpg]

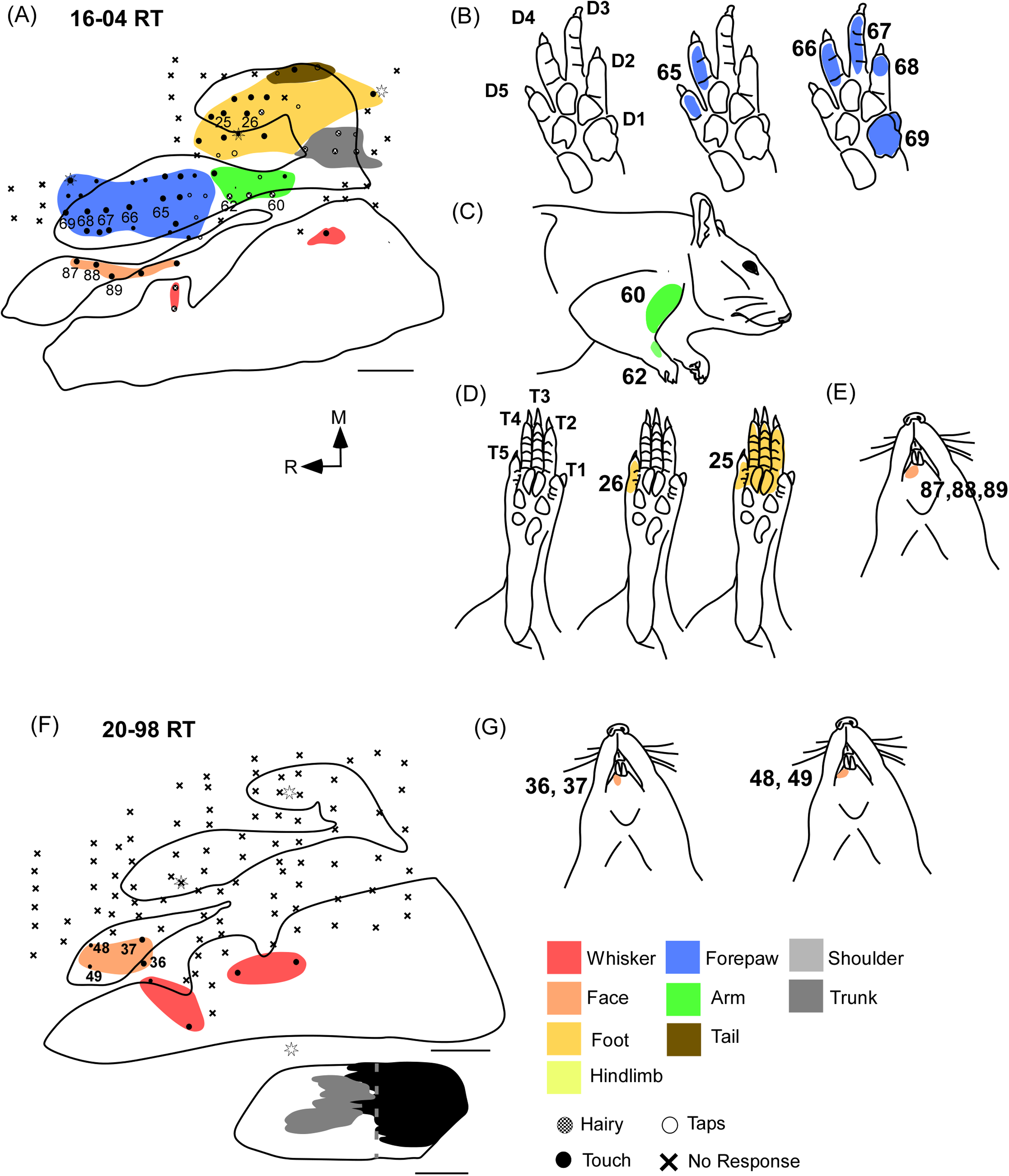

Supplement: Supplementary file 4 — Supplementary material [file mmc4.jpg]

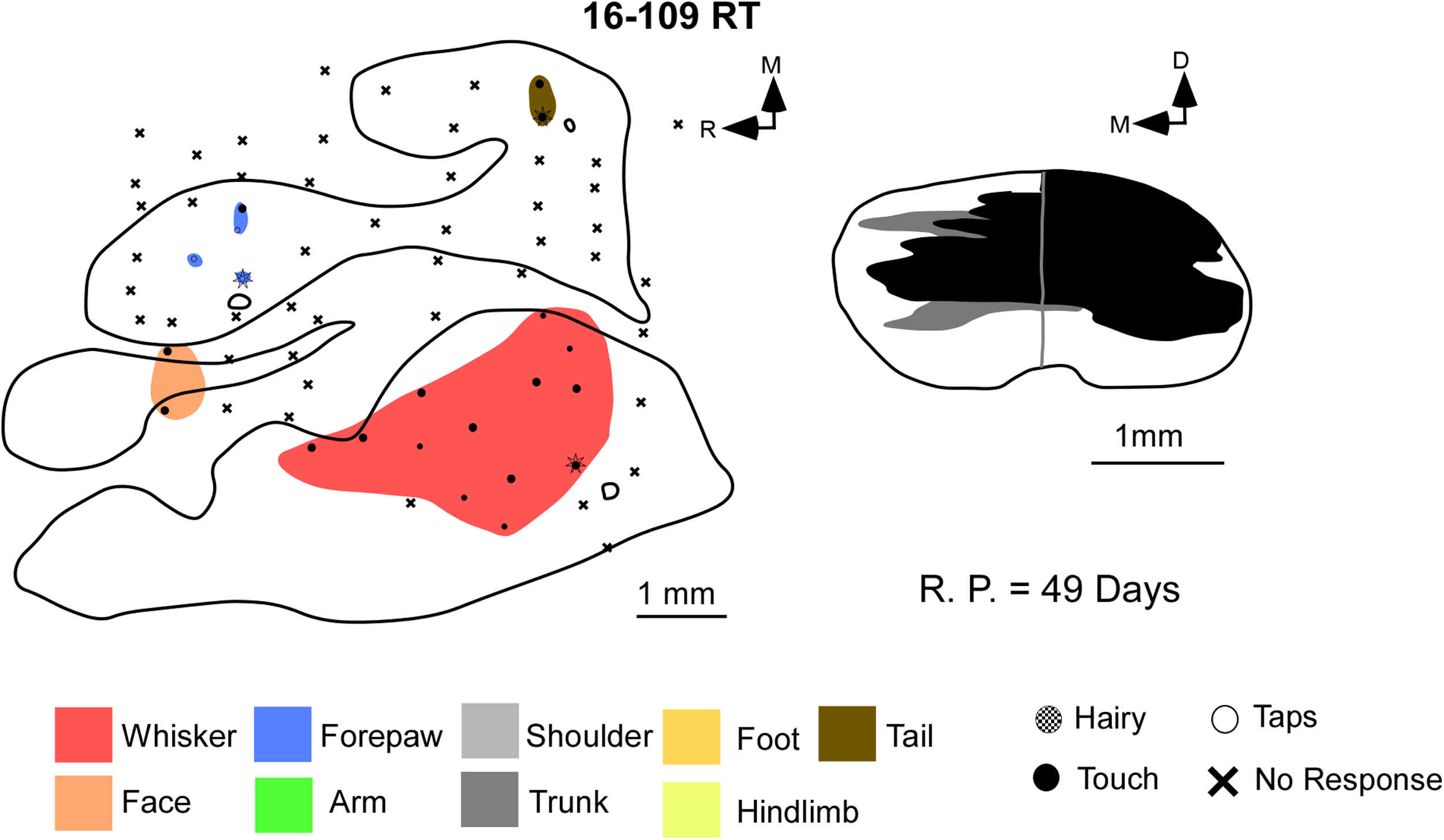

Supplement: Supplementary file 5 — Supplementary material [file mmc5.jpg]

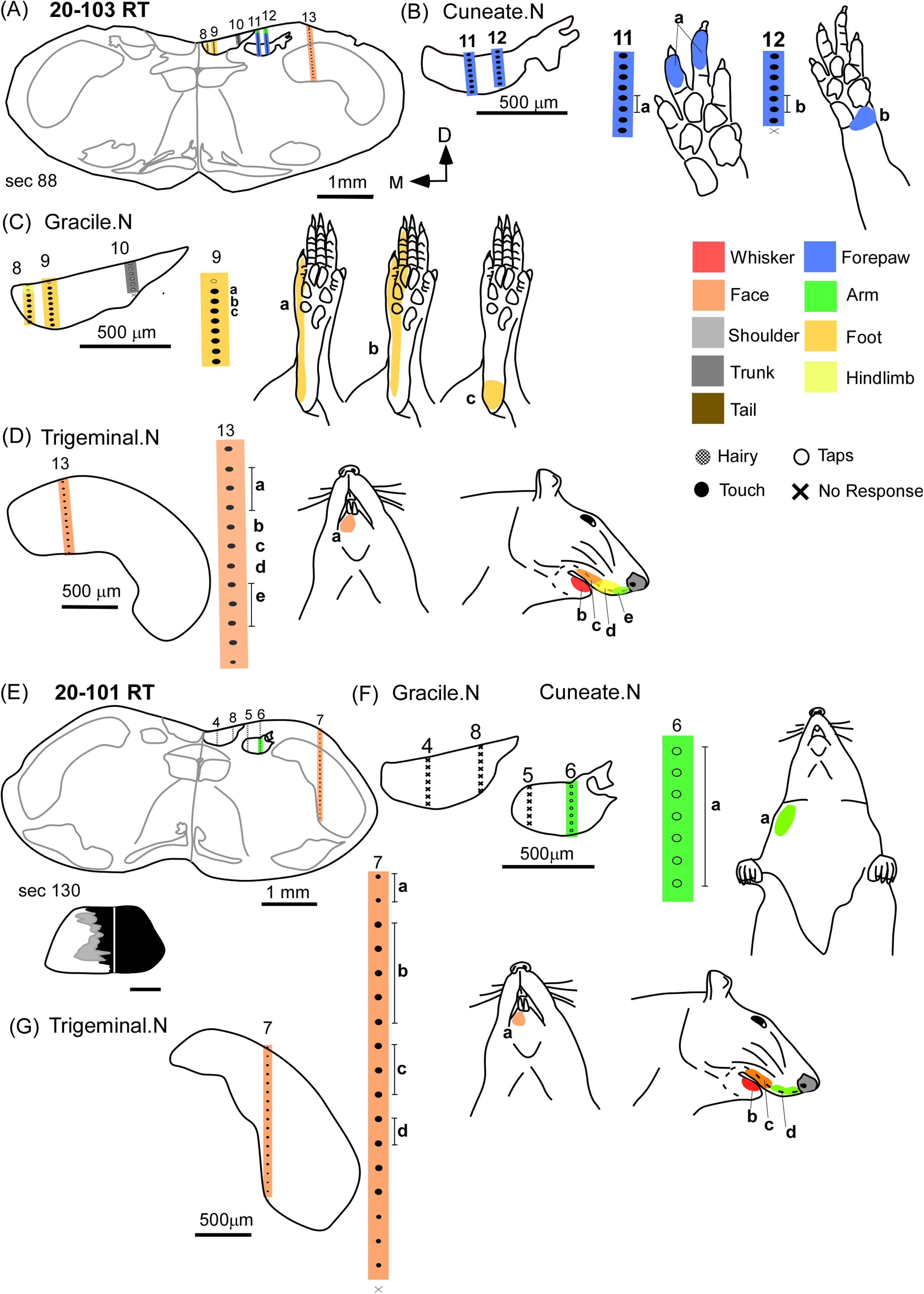

Supplement: Supplementary file 6 — Supplementary material [file mmc6.jpg]

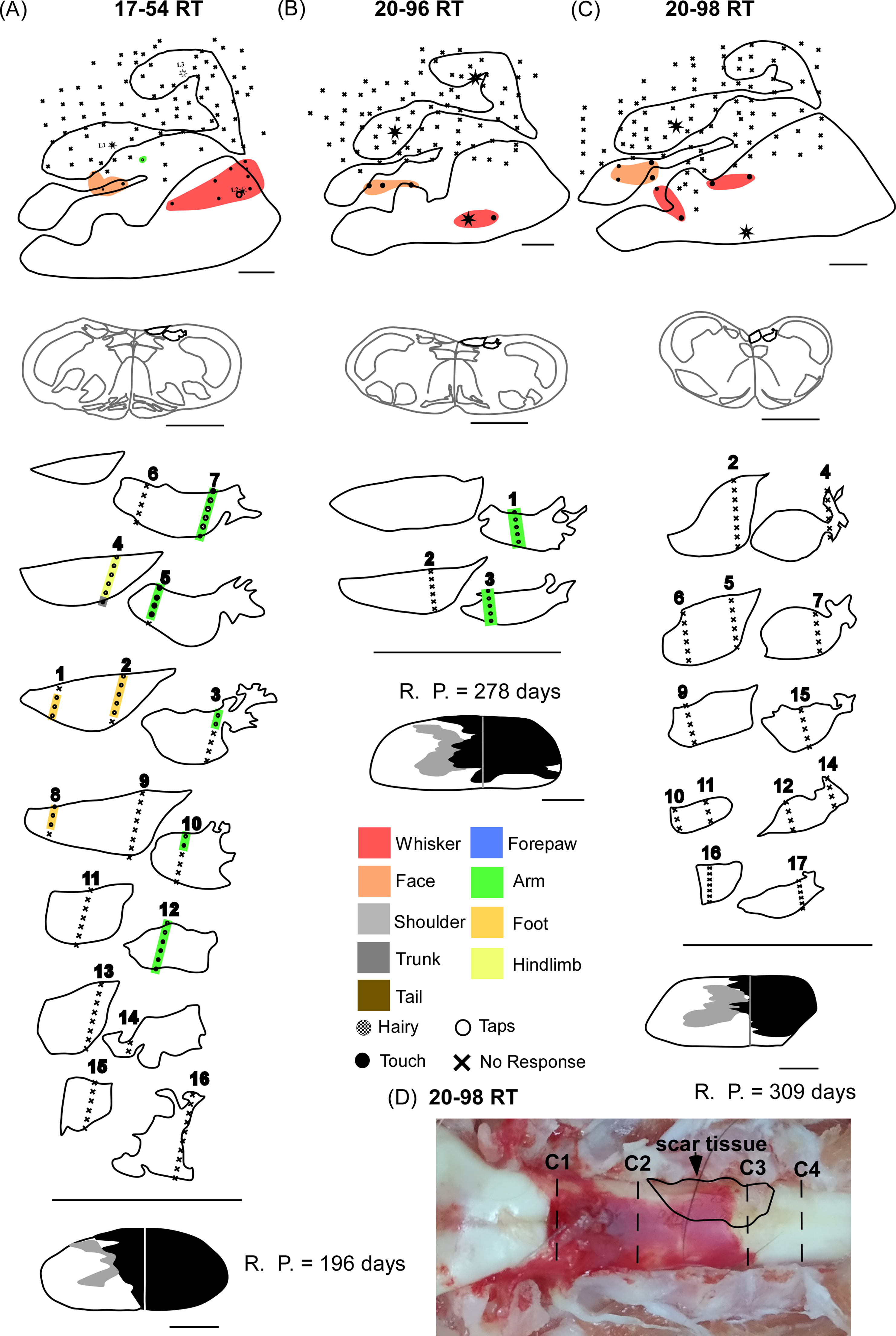

Supplement: Supplementary file 7 — Supplementary material [file mmc7.jpg]
